# The Trypanosome UDP-Glucose Pyrophosphorylase Is Imported by Piggybacking into Glycosomes, Where Unconventional Sugar Nucleotide Synthesis Takes Place

**DOI:** 10.1128/mBio.00375-21

**Published:** 2021-05-28

**Authors:** Oriana Villafraz, Hélène Baudouin, Muriel Mazet, Hanna Kulyk, Jean-William Dupuy, Erika Pineda, Cyrille Botté, Daniel Ken Inaoka, Jean-Charles Portais, Frédéric Bringaud

**Affiliations:** a Université de Bordeaux CNRS, Microbiologie Fondamentale et Pathogénicité (MFP), UMR 5234, Bordeaux, France; b Université de Bordeaux CNRS, Centre de Résonance Magnétique des Systèmes Biologiques (CRMSB), UMR 5536, Bordeaux, France; c Toulouse Biotechnology Institute (TBI), INSA de Toulouse INSA/CNRS 5504, UMR INSA/INRA, Toulouse, France; d MetaboHUB-MetaToul, National Infrastructure of Metabolomics and Fluxomics, Toulouse, France; e Université de Bordeaux Plateforme Protéome, Bordeaux, France; f Apicolipid Team, Institute for Advanced Biosciences, CNRS UMR5309, INSERM U1209, Université Grenoble Alpes, La Tronche, France; g Department of Biomedical Chemistry, Graduate School of Medicine, The University of Tokyo, Tokyo, Japan; h School of Tropical Medicine and Global Health, Nagasaki University, Nagasaki, Japan; i Department of Molecular Infection Dynamics, Shionogi Global Infectious Disease Division, Institute of Tropical Medicine (NEKKEN), Nagasaki University, Nagasaki, Japan; j RESTORE, Université de Toulouse, INSERM U1031, CNRS 5070, Toulouse, France; k EFS, Université Paul Sabatier, Toulouse, France; University of Geneva

**Keywords:** *Trypanosoma brucei*, UDP-glucose pyrophosphorylase, glycosomes, peroxisomes, piggybacking, procyclic form

## Abstract

Glycosomes are peroxisome-related organelles of trypanosomatid parasites containing metabolic pathways, such as glycolysis and biosynthesis of sugar nucleotides, usually present in the cytosol of other eukaryotes. UDP-glucose pyrophosphorylase (UGP), the enzyme responsible for the synthesis of the sugar nucleotide UDP-glucose, is localized in the cytosol and glycosomes of the bloodstream and procyclic trypanosomes, despite the absence of any known peroxisome-targeting signal (PTS1 and PTS2). The questions that we address here are (i) is the unusual glycosomal biosynthetic pathway of sugar nucleotides functional and (ii) how is the PTS-free UGP imported into glycosomes? We showed that UGP is imported into glycosomes by piggybacking on the glycosomal PTS1-containing phosphoenolpyruvate carboxykinase (PEPCK) and identified the domains involved in the UGP/PEPCK interaction. Proximity ligation assays revealed that this interaction occurs in 3 to 10% of glycosomes, suggesting that these correspond to organelles competent for protein import. We also showed that UGP is essential for the growth of trypanosomes and that both the glycosomal and cytosolic metabolic pathways involving UGP are functional, since the lethality of the knockdown UGP mutant cell line (*^RNAi^*UGP, where RNAi indicates RNA interference) was rescued by expressing a recoded UGP (rUGP) in the organelle (*^RNAi^*UGP/*^EXP^*rUGP-GPDH, where GPDH is glycerol-3-phosphate dehydrogenase). Our conclusion was supported by targeted metabolomic analyses (ion chromatography–high-resolution mass spectrometry [IC-HRMS]) showing that UDP-glucose is no longer detectable in the *^RNAi^*UGP mutant, while it is still produced in cells expressing UGP exclusively in the cytosol (PEPCK null mutant) or glycosomes (*^RNAi^*UGP/*^EXP^*rUGP-GPDH). Trypanosomatids are the only known organisms to have selected functional peroxisomal (glycosomal) sugar nucleotide biosynthetic pathways in addition to the canonical cytosolic ones.

## INTRODUCTION

Trypanosoma brucei is a parasite responsible for human African trypanosomiasis, also known as sleeping sickness, a disease affecting sub-Saharan Africa that can be fatal if left untreated (1). This parasite is transmitted through the bite of a tsetse fly and has a complex developmental cycle, including the bloodstream form (BSF) and the procyclic form (PCF) found in the blood of mammalian hosts and the digestive tract of the insect, respectively. A major difference between these two forms is their modes of energy conservation, with the former depending on glucose via glycolysis and the latter being able to use glucose, proline, and other amino acids as carbon sources (2). The complexity of T. brucei’s life cycle leads to the capacity for fast and high adaptation to environmental conditions, mostly through metabolic changes related to energy metabolism. One of the factors playing a role in these efficient changes is the presence of peroxisome-related organelles called glycosomes. The glycosomes contain the first six or seven glycolytic steps, which are commonly present in the cytosol of other eukaryotic cells (3). In addition, the glycosomes contain up to a dozen other metabolic pathways, including the sugar nucleotide biosynthetic pathways, which are also exclusively cytosolic in other organisms (4).

All eukaryotes, excepted trypanosomatids, synthesize sugar nucleotides in the cytosol and then transport them into the lumen of the endoplasmic reticulum (ER) or Golgi apparatus to feed glycosyltransferase-dependent glycosylation reactions (5). In the particular case of trypanosomatids, most of the enzymes involved in *de novo* biosynthesis of sugar nucleotides are present in the glycosomes (6–11). Some of them are known to be essential for the parasite’s survival, probably because the cell surface and endosomal/lysosomal systems are rich in essential glycoconjugates (12).

Within the steps involved in the production of sugar nucleotides, UDP-glucose pyrophosphorylase (UGP) catalyzes the coupling of glucose 1-phosphate (G1P) and UTP to produce UDP-glucose (UDP-Glc) (13). UDP-Glc is a central metabolite that acts as a glucose donor in several pathways, as exemplified by UDP-Glc:glycoprotein glucosyltransferase (UGGT), which uses this sugar nucleotide as a glucosyl donor for protein glycosylation. UDP-Glc has an important role in glycoprotein quality control in the ER, because UGGT specifically glycosylates unfolded glycoproteins to prevent their processing toward the cytosol (14). UDP-Glc is also the obligate precursor of UDP-galactose (UDP-Gal) via a reaction catalyzed by UDP-Glc 4′-epimerase (GalE), given that the parasite hexose transporters are unable to transport galactose (15). The lethality of the T. brucei GalE null mutant makes UDP-Glc production essential for the parasite (9). In the closely related parasites Trypanosoma cruzi and Leishmania major, UDP-sugar pyrophosphorylase (USP) can also activate G1P, in addition to galactose 1-phosphate, while the T. brucei genome does not contain the *USP*-orthologous gene. Consequently, the simultaneous deletion of the *USP* and *UGP* genes is required to deplete the *Leishmania* cells of UDP-Glc and UDP-Gal, leading to growth arrest and cell death (16). In contrast to the animal and fungal UGP, which are octameric (17) and can be regulated by redox mechanisms (18–20) or phosphorylation (21), the characterized T. brucei and L. major UGPs are active as monomers and are regulated by allosteric mechanisms (7, 17, 22).

As recently shown for most of the T. brucei enzymes involved in the biosynthesis of sugar nucleotides, the T. brucei UGP was reported to be localized in glycosomes of BSFs (7, 23). However, it does not contain any of the canonical peroxisomal targeting signals (PTSs) required for import of proteins into the organelle, i.e., the PTS1 tripeptide ([STAGCN]-[RKH]-[LIVMAFY]) or PTS2 ([M]-X_0/20_-[RK]-[LVI]-X_5_-[HQ]-[ILAF], where X refers to any amino acid [with its number in subscript]) located at the C- and N-terminal extremities of the peroxisomal/glycosomal proteins, respectively (24). Alternatively, proteins lacking a PTS can be imported into the organelle by piggybacking through interaction with a PTS-containing protein. The very few examples of piggybacking described so far in peroxisomes of mammals (25, 26), plants (27), and Saccharomyces cerevisiae (28, 29) involve hetero-oligomeric complexes formed by protein isoforms or by functionally related proteins. This mechanism of import has been proposed as an explanation for the presence of some PTS-lacking proteins within glycosomes but has not yet been reported in trypanosomatids so far.

Here, we showed that UGP is imported into glycosomes by interacting with the glycosomal PTS1-containing phosphoenolpyruvate carboxykinase (PEPCK), supporting coimport of functionally unrelated proteins. We also showed that UGP is an essential enzyme for the growth of trypanosomes with dual cytosolic and glycosomal localizations. Metabolomic analyses revealed that UDP-Glc is produced by functional cytosolic and glycosomal pathways. The positive selection of functional sugar nucleotide biosynthesis within glycosomes of trypanosomatids, while this pathway is exclusively cytosolic in other eukaryotes, raises questions about its role in these parasites.

## RESULTS

### UDP-glucose pyrophosphorylase (UGP) has dual glycosomal and cytosolic localizations.

**FIG 1:**
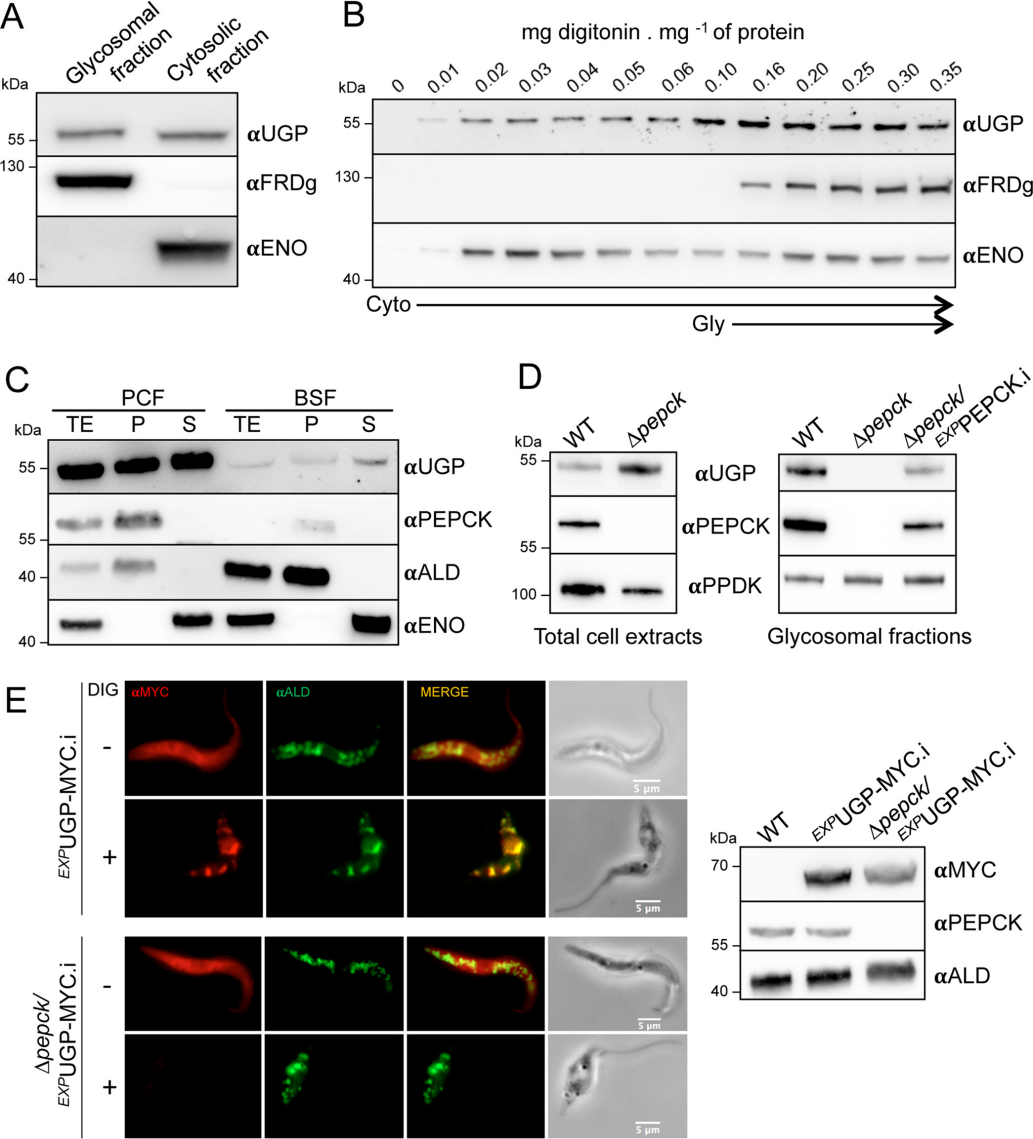
UGP has a dual localization in PCF and BSF, and its import into glycosomes depends on the PTS1-cointaining protein PEPCK. (A and B) Subcellular localization of UGP in the EATRO1125.T7T procyclic trypanosomes. (A) Enriched glycosomal and cytosolic fractions were obtained by differential centrifugation and analyzed by Western blotting using the anti-UGP antibodies (αUGP), as well as immune sera against the glycosomal NADH-dependent fumarate reductase (αFRDg) and cytosolic enolase (αENO) markers. (B) UGP localization was also studied by digitonin titration. The supernatants collected from the parental cells incubated with 0 to 0.35 mg of digitonin per mg of protein were analyzed by Western blotting using the immune sera indicated in the left margin. (C) Comparison of the subcellular localizations and protein expression levels of UGP and PEPCK, as well as the aldolase glycosomal and enolase cytosolic markers, in PCF and BSF trypanosomes. Total extracts (TE), pellets (P), and supernatants (S) obtained after hypotonic lysis were analyzed by Western blotting using the immune sera indicated. (D) Western blot analysis of total cellular extracts and glycosomal fractions of the WT, the Δ*pepck* null mutant, and the tetracycline-induced (.i) Δ*pepck*/*^EXP^*PEPCK rescue cell line (Δ*pepck*/*^EXP^*PEPCK.i) using the anti-UGP, anti-PEPCK, and anti-PPDK (anti-pyruvate phosphate dikinase) immune sera. (E, left) the UGP subcellular localization was analyzed by immunofluorescence of cell lines expressing a recombinant MYC-tagged UGP in the WT (*^EXP^*UGP-MYC cell line) and Δ*pepck* (Δ*pepck*/*^EXP^*UGP-MYC) backgrounds, using anti-MYC (red) and the glycosomal ALD (green) control. Before fixation, the cells were pretreated with 0.04 mg digitonin (DIG)/mg of protein (+) to remove the cytosolic UGP-MYC signal or not treated (–). The expression of UGP-MYC was confirmed by Western blotting of total cell extracts (right) using anti-MYC, anti-PEPCK, and anti-ALD as loading controls.

Previous studies on the UGP subcellular localization revealed that the protein is associated with glycosomes of the BSF (7), despite the absence of any predicted peroxisomal targeting signal (PTS1/PTS2). We raised an anti-UGP (αUGP) immune serum to confirm this unique glycosomal localization of UGP in the PCF by Western blotting of glycosomal and cytosolic fractions prepared by differential centrifugation, using control antibodies against glycosomal (NADH-dependent fumarate reductase [FRDg]) and cytosolic (enolase [ENO]) proteins. The anti-UGP immune serum detected a 55-kDa protein corresponding to the predicted size of UGP (theoretical molecular weight [MW], 54.5 kDa) in both the glycosomal and cytosolic fractions (Fig. 1A). This dual localization was further confirmed by digitonin titration, as UGP was released together with the cytosolic protein at low concentrations of detergent and the UGP signal increased with the digitonin concentration required to release the glycosomal marker (Fig. 1B). The increased signal at higher digitonin concentrations suggests that the total amount of UGP in the glycosomes is at least equivalent to that in the cytosol. We also addressed the UGP subcellular localization in BSFs by performing hypotonic lysis, which released cytosolic proteins, while glycosomal proteins remained in the cellular pellet, as evidenced by the glycosomal aldolase and cytosolic enolase markers (Fig. 1C). UGP is similarly distributed over the two compartments in BSFs, as observed for PCFs (Fig. 1C).

### PEPCK-dependent import of UGP into glycosomes.

Incidentally, a comparative proteomic analysis of the previously obtained PEPCK null (Δ*pepck*) mutant (30) and the parental cell line, carried out in order to control the *PEPCK* gene deletion, showed a strong reduction (19.7-fold) of UGP peptide counts in the enriched glycosomal fractions of the mutant (see the PXD020190 data set in the PRIDE partner repository). Depletion of UGP in the glycosomes of the Δ*pepck* cell line was confirmed by Western blotting, showing that UGP was no longer detected in the Δ*pepck* glycosomes, while the protein was still present in the total cell extracts (Fig. 1D). Importantly, reexpression of the *PEPCK* gene in the *PEPCK* null background (Δ*pepck*/*^EXP^*PEPCK.i cell line [“*EXP*” stands for “expressing,” and “.i” stands for tetracycline-induced]) rescued the glycosomal localization of UGP (Fig. 1D). These data suggest that import of UGP into the glycosomes depends on the presence of PTS1-containing PEPCK, potentially by the so-called piggybacking mechanism not reported so far in trypanosomatids (31). In this context, UGP would be cotransported with PEPCK, which is imported into the glycosome via its PTS1.

To confirm the dual subcellular localization of UGP, we produced cell lines expressing a MYC-tagged UGP under the control of tetracycline in both the parental and the Δ*pepck* backgrounds (Fig. 1E, right panel). Immunofluorescence analyses showed a clear cytosolic pattern in the tetracycline-induced *^EXP^*UGP-MYC.i and Δ*pepck*/*^EXP^*UGP-MYC.i cell lines (Fig. 1E, left panel). A signal colocalizing with the glycosomal marker aldolase was detected for the *^EXP^*UGP-MYC.i cells only after pretreatment with 0.04 mg of digitonin per mg of protein required for permeabilization of the plasma membrane. These data confirmed that recombinant UGP-MYC exhibits dual localizations, similar to that in the native protein. Interestingly, the glycosomal signal was not detected in the Δ*pepck*/UGP-MYC.i cell line after digitonin treatment, indicating that all UGP localizes exclusively in the cytosol of this mutant. Altogether, these data support the role of PEPCK in the import of UGP into glycosomes.

### UGP interacts with PEPCK in some glycosomes.

To evidence the putative interaction between UGP and PEPCK, we used proximity ligation assays (PLA; Duolink), which enable detection of protein interactions, including transient/weak interactions *in situ*, with high specificity and sensitivity (32). We produced a Δ*pepck*/*^EXP^*TY-PEPCK/*^EXP^*UGP-MYC cell line expressing TY-tagged PEPCK (TY-PEPCK; TY stands for the Ty1 epitope: EVHTNQDPLD) and MYC-tagged UGP (UGP-MYC) in the *PEPCK* null background. Briefly, the second *PEPCK* allele of the single-allele Δ*pepck/*PEPCK knockout cell line was replaced by a *TY-PEPCK* copy encoding TY-PEPCK tagged at its N-terminal extremity to preserve the PTS1 motif required for glycosomal import. This Δ*pepck*/*^EXP^*TY-PEPCK cell line was transfected with the pLew100-*^EXP^*UGP-MYC plasmid to express UGP-MYC under the control of tetracycline. As controls, the UGP-MYC and TY-PEPCK recombinant proteins have been independently expressed in the Δ*pepck* (Δ*pepck*/*^EXP^*UGP-MYC) and parental (*^EXP^*TY-PEPCK) backgrounds, respectively. The expression of both recombinant proteins, the specificity of the primary antibodies, and the glycosomal import of TY-PEPCK and UGP-MYC were confirmed by Western blotting analyses of enriched glycosomal and cytosolic fractions, digitonin titration, and immunofluorescence analyses (see Fig. S1 in the supplemental material). As expected, TY-PEPCK showed a glycosomal localization; however, its level of expression was ∼8 times lower than that of the native protein (Fig. S1A, compare the upper and lower bands of the αPEPCK signal in the *^EXP^*TY-PEPCK cell line, respectively). Despite this difference in expression levels, a significant part of the recombinant UGP-MYC is imported into the glycosomes of the tetracycline-induced Δ*pepck*/*^EXP^*TY-PEPCK/*^EXP^*UGP-MYC.i cell line (Fig. S1A and B), while remaining exclusively in the cytosol of the Δ*pepck*/*^EXP^*UGP-MYC.i cell line (Fig. S1A), as previously shown (Fig. 1E). αMYC (rabbit) and αTY (mouse) were validated to be specific and sensitive enough for us to perform PLA analysis (Fig. S1C).

10.1128/mBio.00375-21.1 FIG S1 Analysis of tagged UGP-MYC and TY-PEPCK cell lines. (A) Western blot analysis of enriched glycosomal (G) and cytosolic (C) fractions obtained by differential centrifugation. Anti-PEPCK (αPEPCK) recognizes tagged TY-PEPCK in addition to native PEPCK. The samples were also analyzed with the anti-TY (αTY) and anti-MYC (αMYC) markers, as well as with the glycosomal (αPPDK) and cytosolic (αENO) markers. (B) Digitonin titration analysis of the Δ*pepck*/*^EXP^*TY-PEPCK/*^EXP^*UGP-MYC.i cell line. Western blot analyses of pellets confirmed the glycosomal and cytosolic localizations of recombinant UGP-MYC with anti-MYC antibodies. (C) Subcellular localization of tagged UGP-MYC and TY-PEPCK proteins in the Δ*pepck*/*^EXP^*TY-PEPCK/*^EXP^*UGP-MYC, Δ*pepck*/*^EXP^*UGP-MYC, and *^EXP^*TY-PEPCK cell lines by immunofluorescence microscopy analyses using rabbit anti-MYC and mouse anti-TY antibodies. Download FIG S1, TIF file, 0.3 MB. Copyright © 2021 Villafraz et al. 2021 Villafraz et al. https://creativecommons.org/licenses/by/4.0/ This content is distributed under the terms of the Creative Commons Attribution 4.0 International license.

**FIG 2:**
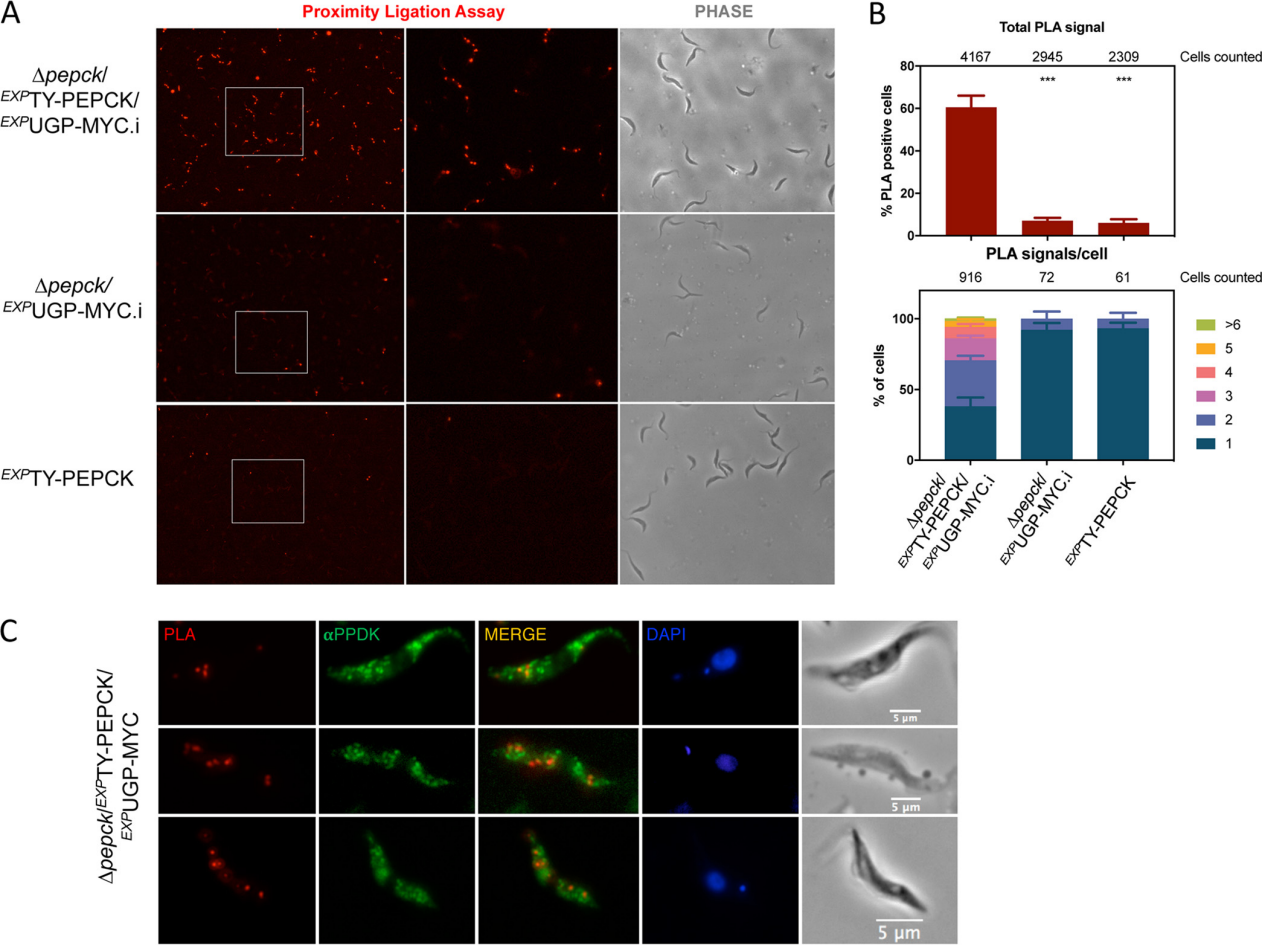
UGP interacts transiently with PEPCK. (A) *In situ* proximity ligation assay (PLA) analysis of the interaction between MYC-tagged UGP and TY-tagged PEPCK in the Δ*pepck*/*^EXP^*TY-PEPCK/*^EXP^*UGP-MYC.i cell line. The *^EXP^*TY-PEPCK and Δ*pepck*/*^EXP^*UGP-MYC.i cell lines expressing only one recombinant protein were used as negative controls. The central (PLA) and right (phase contrast) panels are enlargements of the white rectangles shown in the left panel. (B) The percentage of PLA-positive cells is shown for each cell line, and the total cell number counted is indicated on the top of the graph. The percentages correspond to averages of 12 pictures randomly taken from 2 independent experiments. Significant differences between samples are indicated: ***, *P < *0.001. The number of PLA signals per cell was analyzed by counting manually the number of dots using ImageJ for positive cells (lower panel). (C) The localization of the PLA signal was analyzed in detail and compared with that of the PPDK glycosomal marker (αPPDK) by counterstaining after the Duolink *in situ* protocol.

PLA-positive puncta (red signals) corresponding to TY-PEPCK/UGP-MYC hetero-oligomers were observed in 62% of the Δ*pepck*/*^EXP^*TY-PEPCK/*^EXP^*UGP-MYC.i cells, while only 7% and 6% of the control Δ*pepck*/*^EXP^*UGP-MYC.i and *^EXP^*TY-PEPCK.i cells were positive, respectively (Fig. 2A and B). In addition, ∼90% of the positive Δ*pepck*/*^EXP^*UGP-MYC.i and *^EXP^*TY-PEPCK.i cells contained a single dot, and the other 10% contained 2 dots, while the number of dots per cell in the Δ*pepck*/*^EXP^*TY-PEPCK/*^EXP^*UGP-MYC.i population was much higher, with 62% of the cells showing 2 to 10 dots (Fig. 2B). These data are in agreement with interactions between UGP-MYC and TY-PEPCK in Δ*pepck*/*^EXP^*TY-PEPCK/*^EXP^*UGP-MYC.i cells, while the very few red dots observed within the control Δ*pepck*/*^EXP^*UGP-MYC.i and *^EXP^*TY-PEPCK.i cells represent background signals. Staining with an immune serum against the glycosomal PPDK showed that the PLA signals are found very close to the PPDK-containing organelles, without showing clear colocalization with them (Fig. 2C). This suggests the existence of different pools of glycosomes, as previously reported (33).

### Determination of critical parts for PEPCK-UGP interaction.

**FIG 3:**
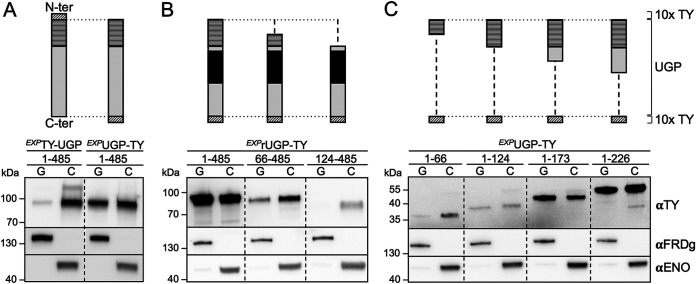
The 123 N-terminal residues of UGP are required for import into the glycosomes. The top of the figure shows schematic representations of the 10×TY-tagged native or recoded UGP and the corresponding truncations. The 123-residue peptide required for UGP import, the recoded part of UGP, and the 10×TY tags are highlighted by horizontally striped boxes, black boxes, and obliquely hatched boxes, respectively. The lower part of the figure shows results of Western blot analyses of glycosomal (G) and cytosolic (C) fractions produced from cell lines expressing 10×TY-tagged recombinant UGP using the anti-TY antibody (αTY), as well as immune sera from glycosomal (αFRDg) and cytosolic (αENO) markers. (A) Recombinant UGP proteins tagged at their N-terminal (N-ter) (*^EXP^*TY-UGP_1–485_) or C-terminal (C-ter) (*^EXP^*UGP_1–485_-TY) extremities; (B and C) truncated UGP tagged at its C-terminal extremity. The truncations designed from the N terminus lack the first 65 (66 to 485) or 123 (124 to 485) residues (B), while truncations designed from the C terminus contain the first 66 (1 to 66), 124 (1 to 124), 173 (1 to 173), or 226 (1 to 226) N-terminal residues (C). A PCR analysis was performed to confirm the correct insertion of the UGP_XXX–485_-TY fragments at the *UGP* locus (see Fig. S3 in the supplemental material).

To investigate which part of UGP and PEPCK interacts with its piggybacking partner, truncated versions of each protein were expressed in the parental and Δ*pepck* cell lines, respectively. Since PEPCK forms homodimers (34), the truncated PEPCK proteins were expressed in the Δ*pepck* cell line to prevent heterodimer formation. UGP is reported to be monomeric (7, 22) and was detected only as monomer in native gel analyses (Fig. S2); therefore, the native and recombinant proteins will not directly interact. We expressed in the parental background the recombinant UGP with the 10×TY tag either at the N-terminal or the C-terminal end of UGP (*^EXP^*TY-UGP_1–485_ and *^EXP^*UGP_1–485_-TY cell lines, respectively) by *in situ* replacement of one *UGP* allele. The subcellular distribution of UGP in these cell lines was determined by Western blotting of glycosomal and cytosolic fractions. The N-terminal tag affected the glycosomal import of UGP, since the glycosomal localization of TY-UGP_1–485_ was decreased by ∼9-fold compared to that of the native UGP in parental cells (Fig. 3A, left panel). However, no changes were observed in the glycosome/cytosol ratio for UGP_1–485_-TY (Fig. 3A, right panel, compared with Fig. 1A). C-terminally tagged recombinant UGP versions truncated from their N-terminal (UGP_XXX–485_-TY) (Fig. 3B) or C-terminal (UGP_1–XXX_-TY) (Fig. 3C) extremities were inserted *in situ* to produce new cell lines. It is useful to note that the UGP coding sequence used for the UGP_XXX-485_-TY constructs was recoded from amino acid positions 165 to 337 to become resistant to the RNA interference (RNAi) construct (see below), which was useful to confirm the correct insertion of the recombinant fragment in the *UGP* locus (Fig. S3). The truncated recoded UGP protein with amino acids 124 to 485 (rUGP_124–485_)-TY was no longer imported into glycosomes (Fig. 3B), while glycosomal import of the UGP_1–124_-TY, UGP_1–173_-TY, and UGP_1–226_-TY proteins was not affected (Fig. 3C), suggesting that the N-terminal domain up to amino acid position 123 contains residues interacting with PEPCK. The truncated recombinant UGP missing (rUGP_66–485_-TY) or containing (UGP_1–66_-TY) only the 66 N-terminal residues were imported into glycosomes, although with a lower efficiency than occurred with the parental cell line, suggesting that key residues of the PEPCK binding site are located on either side of position 66 (Fig. 3B and C). The presence of the PEPCK binding site in the N-terminal extremity of UGP may explain the low glycosomal import of the recombinant TY-UGP_1–485_ protein (Fig. 3A).

10.1128/mBio.00375-21.2 FIG S2 Analysis of UGP oligomerization in a native gel. The oligomeric state of tagged UGP-MYC was evaluated in the *^EXP^*UGP-MYC.i cell line. Supernatants obtained after digitonin treatment (0.16 mg of digitonin per mg of protein) or no digitonin treatment (0) were analyzed by BN-PAGE (left) and Western blotting (right) using the anti-MYC antibody. Download FIG S2, TIF file, 0.9 MB. Copyright © 2021 Villafraz et al. 2021 Villafraz et al. https://creativecommons.org/licenses/by/4.0/ This content is distributed under the terms of the Creative Commons Attribution 4.0 International license.

10.1128/mBio.00375-21.3 FIG S3 PCR analysis to confirm endo- tagging at the *UGP* locus. (A) Schematic representation of the *UGP* native and tagged alleles in the WT, *^EXP^*rUGP_1–485_-TY, *^EXP^*rUGP_66-485_-TY, and *^EXP^*rUGP_164-485_-TY cell lines. The primers were designed to amplify products to differentiate between the native (product 1) and the recoded (product 2) tagged alleles. The recoded region (rUGP) is indicated by black boxes. PCR product 3 confirms the TY tagging at the C terminus. (B) PCR analysis of products 1, 2, and 3 using genomic DNA from parental cells as a control. Download FIG S3, TIF file, 0.7 MB. Copyright © 2021 Villafraz et al. 2021 Villafraz et al. https://creativecommons.org/licenses/by/4.0/ This content is distributed under the terms of the Creative Commons Attribution 4.0 International license.

**FIG 4:**
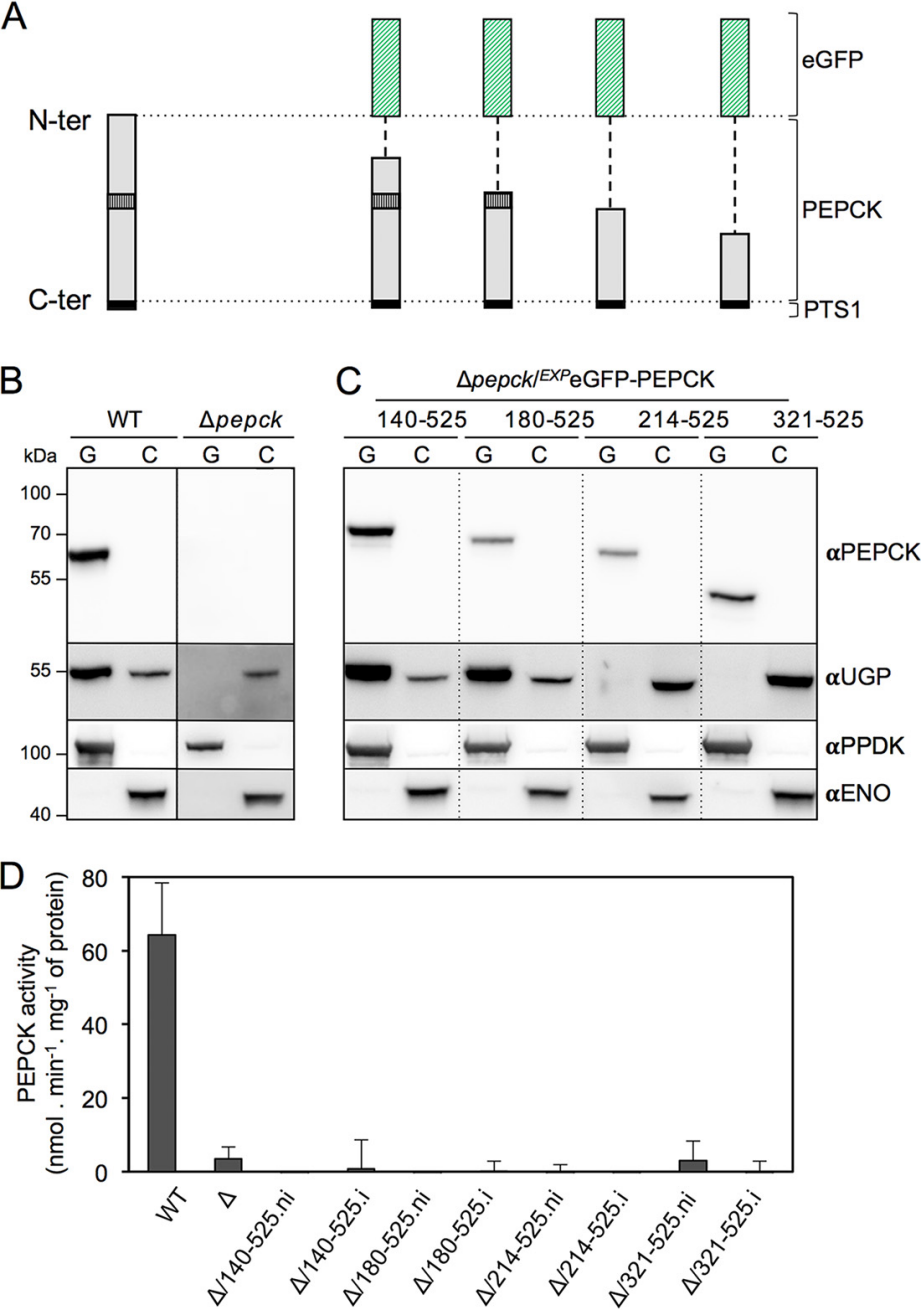
A 34-residue peptide of PEPCK is required for glycosomal import of UGP. (A) Schematic representations of the endogenous PEPCK and eGFP-PEPCK recombinant proteins expressed in the Δ*pepck* background, in which the 34-residue peptide required for UGP import (vertically striped boxes) is highlighted. (B and C) Western blot analyses of glycosomal (G) and cytosolic (C) fractions obtained from the parental and Δ*pepck* cell lines (B), as well as from cell lines expressing truncated recombinant eGFP-PEPCK proteins (Δ*pepck*/*^EXP^*eGFP-PEPCK_XXX–525_) with anti-PEPCK and anti-UGP immune sera. Glycosomal (αPPDK) and cytosolic (αENO) markers are used to check the quality of glycosomal and cytosolic fractions. (D) PEPCK activity determined in total extracts of the WT, the Δ*pepck* (Δ), and the noninduced (.ni) and induced (.i) Δ*pepck*/*^EXP^*eGFP-PEPCK_XXX–525_ cell lines.

We performed a similar analysis to determine the PEPCK region involved in UGP glycosomal import by expressing truncated versions of recombinant PEPCK using the pLew100 vector. PEPCK was truncated from its N-terminal extremity in order to maintain C-terminal PTS1, required for glycosomal import of both PEPCK and UGP. Unfortunately, none of the truncated PEPCK peptides were detectable by Western blotting in total cell extracts, probably due to protein instability. To resolve this stability issue, the truncated PEPCK peptides were fused to the C-terminal extremity of the enhanced green fluorescent protein (eGFP) and used to produce four different cell lines (Fig. 4A). We determined the glycosomal import of UGP in these Δ*pepck*/*^EXP^*eGFP-PEPCK_XXX–525_ cell lines by Western blotting of glycosomal and cytosolic fractions. As mentioned above, UGP was no longer detected in glycosomes isolated from the parental Δ*pepck* mutants (Fig. 4B). The glycosomal import of UGP was not affected in the absence of the first 140 and 180 N-terminal residues of PEPCK (Δ*pepck*/*^EXP^*eGFP-PEPCK_140–525_ and Δ*pepck*/*^EXP^*eGFP-PEPCK_180–525_ cell lines), while deletion of the first 214 and 321 N-terminal residues abolished the glycosomal import of UGP, which remained exclusively in the cytosolic fractions (Fig. 4C). This suggested that the 34-residue peptide between amino acids positions 180 and 214 of PEPCK is required for UGP import into glycosomes. Importantly, none of the eGFP-PEPCK truncations have PEPCK activity, indicating that the import of UGP is not related to PEPCK activity inside the glycosomes (Fig. 4D).

### Targeting a recombinant UGP exclusively to the glycosomes.

**FIG 5:**
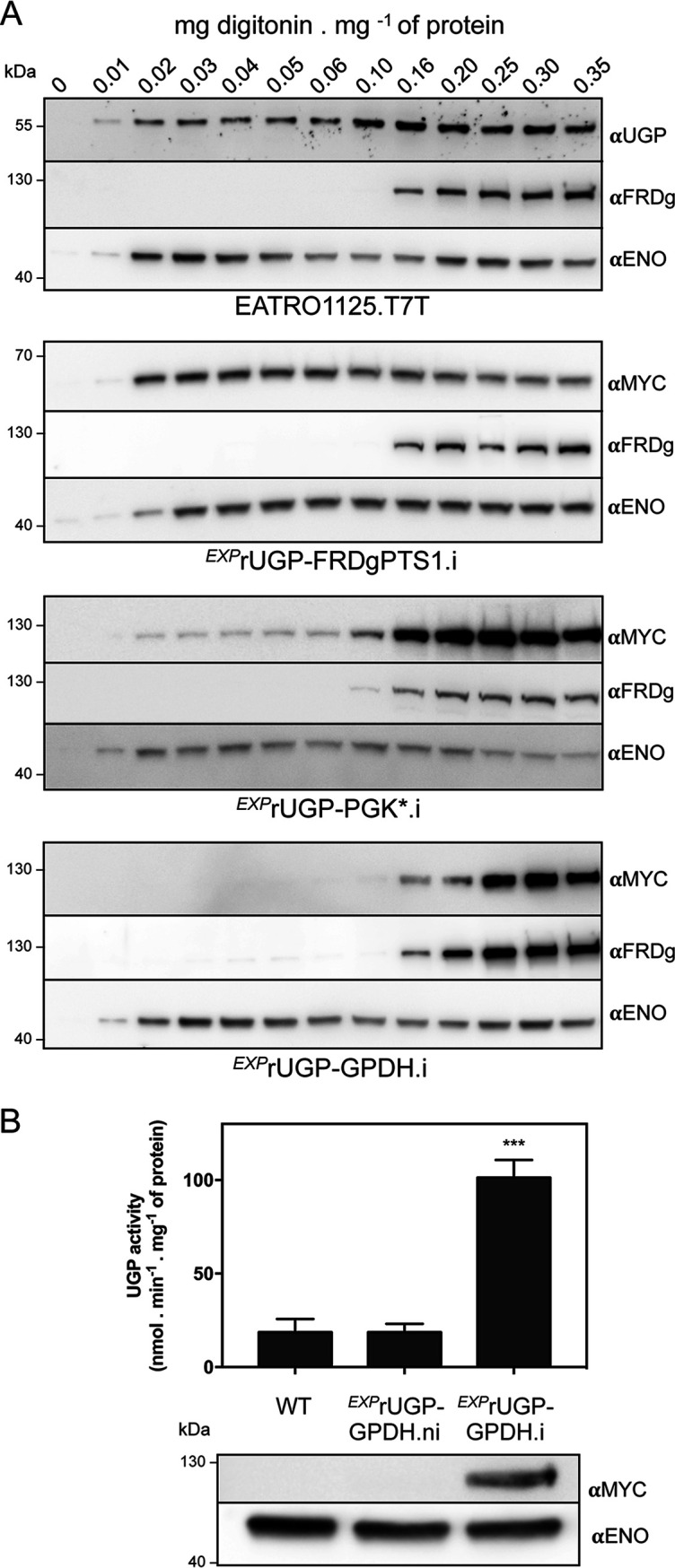
Expression of a glycosomal recombinant UGP. (A) The subcellular localization of recombinant UGP was monitored by Western blotting of supernatants obtained after digitonin titration of the tetracycline-induced *^EXP^*rUGP-FRDgPTS1, *^EXP^*rUGP-PGKc*, and *^EXP^*rUGP-GPDH cell lines using anti-MYC antibody. (Top) The anti-UGP immune serum was used to detect UGP in parental cells fractions. Anti-FRDg and anti-ENO immune sera were used as glycosomal and cytosolic markers, respectively. (B) UGP activity measured in total cell extracts of parental (WT) and tetracycline-induced and noninduced *^EXP^*rUGP-GPDH cell lines (*n *= 3; standard errors of the means [SEM]). Significant differences between samples are indicated. ***, *P < *0.001. (Bottom) A Western blot analysis of *^EXP^*rUGP-GPDH expression with anti-MYC and anti-ENO (loading control) immune sera is shown below the graph.

To elucidate in which subcellular compartment the UDP-Glc/UDP-Gal biosynthetic pathway is active (glycosomes and/or cytosol), it was necessary to express UGP exclusively in the cytosol or in the glycosomes of the parasite. The exclusive cytosolic localization of UGP in the viable Δ*pepck* mutant demonstrated that UGP is functionally active in the cytosol. To assess the role of UGP in glycosomes, we optimized the glycosomal import of UGP with the objective that all of the recombinant UGP is localized within the glycosomes. To do so, a recombinant *UGP* gene recoded to become resistant to the RNAi construct (*rUGP*) was fused at its 3′ extremity with a 3×MYC tag followed by different glycosomal targeting peptides (PTS1), namely, the last 12 C-terminal residues of glycosomal FRDg (*rUGP-FRDgPTS1*), the full-length PTS1-containing glycosomal glycerol-3-phosphate dehydrogenase (*GPDH*) gene (*rUGP-GPDH*), and the full-length PTS1-containing glycosomal phosphoglycerate (*PGKc*) gene (rUGP-PGKc*). Since glycosomal expression of PGK is lethal for the PCF trypanosomes (35), the codon of the lysine residue (K215) essential for the PGK enzymatic activity (36) was replaced by the alanine codon. These recombinant proteins were conditionally expressed in the parental cell line, and their distribution between the glycosomal and cytosolic compartments was determined by digitonin titration (Fig. 5A). The rUGP-FRDgPTS1 and cytosolic enolase proteins showed the same cytosolic profiles, which implies that the extended FRDg PST1 motif is not sufficient for glycosomal import of UGP. In contrast, the rUGP-PGKc* recombinant protein is mostly associated with the glycosomes, but a minor part remained in the cytosol. Finally, both the rUGP-GPDH (∼100-kDa) and the glycosomal FRDg proteins were released with a minimum of 0.16 mg digitonin per mg protein, which is consistent with the exclusively glycosomal localization of this recombinant protein. The UGP activity was increased by 5-fold in the *^EXP^*rUGP-GPDH.i cell line compared to those in the noninduced (.ni) and parental cell lines, which validated the functionality of the rUGP-GPDH protein (Fig. 5B). Expression of rUGP-GPDH had no effect on the morphology, growth, or survival of the *^EXP^*rUGP-GPDH cell line.

### The UGP protein is essential for T. brucei.

**FIG 6:**
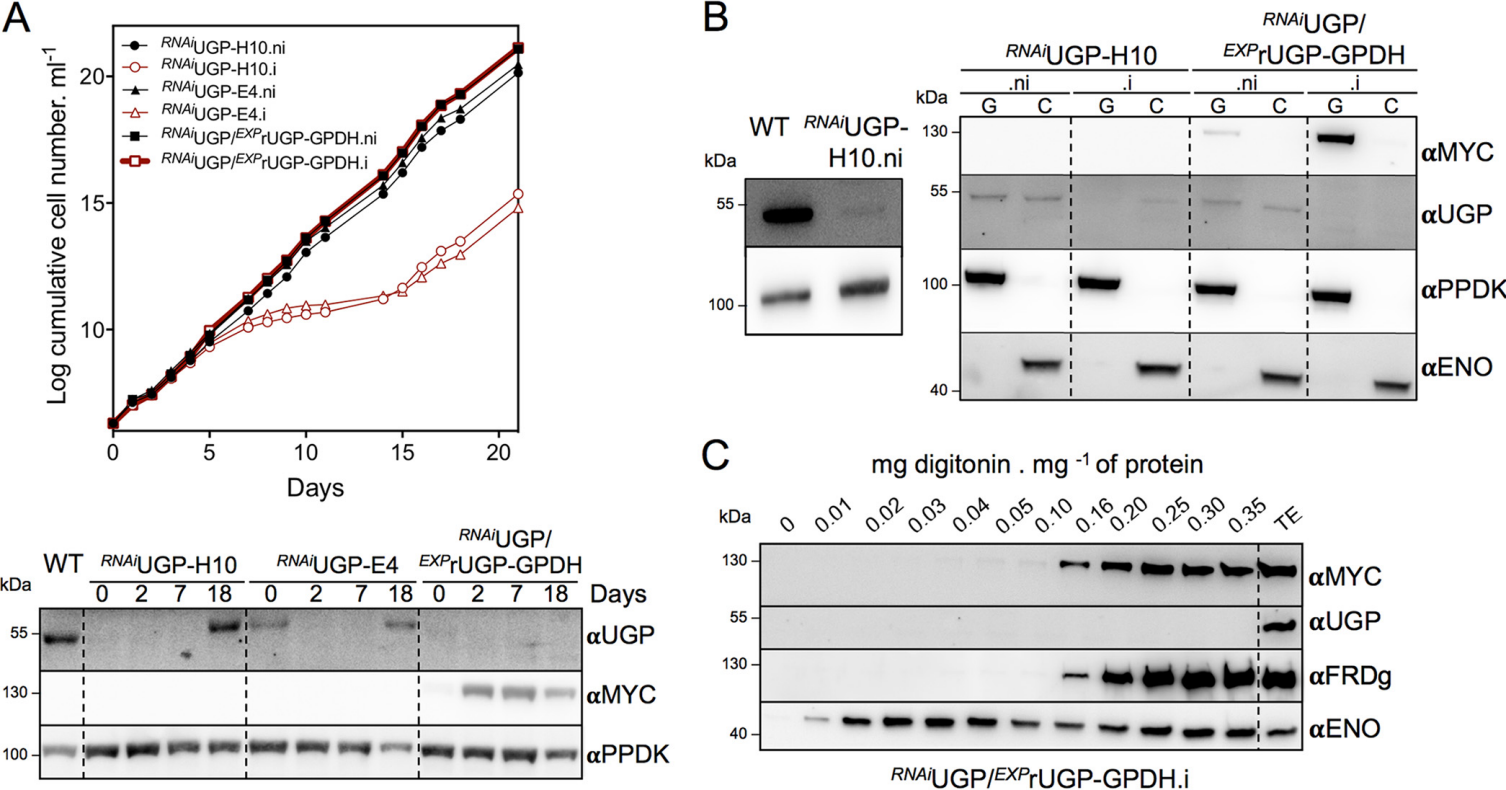
Production and functional analyses of *^RNAi^*UGP cell lines. (A) Growth curve of the tetracycline-induced and noninduced *^RNAi^*UGP-H10, *^RNAi^*UGP-E4, and *^RNAi^*UGP/*^EXP^*rUGP-GPDH cell lines. The expression of native UGP and recombinant rUGP-GPDH upon induction was monitored by Western blotting using anti-UGP and anti-MYC immune sera, respectively, and anti-PPDK as a loading control (bottom). (B) Western blot analyses of glycosomal (G) and cytosolic (C) fractions produced from the parental cells (WT), as well as the tetracycline-induced and noninduced *^RNA^*^i^UGP-H10 and *^RNAi^*UGP/*^EXP^*rUGP-GPDH cell lines, using immune sera described in the preceding figures. (Left) Quantification of the relative expression of UGP in glycosomes of the noninduced *^RNA^*^i^UGP-H10 mutant. (C) The glycosomal localization of recombinant rUGP-GPDH (αMYC) was confirmed by Western blotting analyses of supernatants obtained after digitonin titration of the *^RNAi^*UGP/*^EXP^*rUGP-GPDH.i cell line, using the immune sera described in precedent figures. The control lane TE corresponds to total extract from the *^EXP^*rUGP-GPDH.i cell line.

The stem-loop RNAi strategy was used with the conditional pLew100 vector to address the role of UGP in the procyclic trypanosomes. Two *^RNAi^*UGP cell lines obtained from individual transfections (H10 and E4) showed a strong reduction of growth 7 days after tetracycline induction, indicating that UGP is essential for PCF viability (Fig. 6A, top panel). For both RNAi cell lines, the growth rate of the parental strain was restored 18 days postinduction, concomitantly with the reexpression of the native UGP (Fig. 6A, lower panel). This reexpression of RNAi-targeted genes is often observed for trypanosome essential genes (30). It is noteworthy that the UGP expression was barely detectable in the noninduced *^RNAi^*UGP-H10 total cell extracts. Western blot analyses of enriched glycosomal fractions, which proved to be more sensitive than on total cell extracts, showed that UGP expression was reduced by ∼30-fold compared to that in the parental cells, without any significant effect on growth (Fig. 6B, left panel). This suggests that UGP activity is present in large excess in parental PCFs. The distribution of UGP between glycosomal and cytosolic compartments is not affected by this ∼30-fold reduction (Fig. 6B). After 5 days of induction, UGP was no longer detectable in the glycosomal fractions and was reduced by ∼2-fold in the cytosol (Fig. 6B). These small amounts of UGP were not sufficient to sustain the growth of PCFs.

To determine whether UGP is also required for the growth of the procyclic trypanosomes under the insect-like glucose-free conditions, the parasites were grown in the absence of glucose, as described before (37). The growth of the *^RNAi^*UGP.i and Δ*pepck*/*^RNAi^*UGP.i cell lines is similar regardless of the amounts of glucose in the medium (Fig. S4), indicating that the UGP is probably also essential in the insect vector, which is considered to be free of glucose (38). In addition, the subcellular distribution of UGP in the parental cells is not affected by the absence of glucose (Fig. S5).

10.1128/mBio.00375-21.4 FIG S4 Functional analysis of *^RNAi^*UGP cell lines in the presence or absence of glucose. Growth curves of the tetracycline-induced and noninduced *^RNAi^*UGP and Δ*pepck*/*^RNAi^*UGP cell lines. The cells were grown in SDM79-GlcFree medium in the presence of 10 mM glucose (+Glc) or in the absence of glucose with 50 mM *N*-acetyl-glucosamine to inhibit the uptake of residual glucose (–Glu). The expression of PEPCK and UGP was monitored by Western blotting (inset in the right panel), with the immune sera indicated in the right margin. Download FIG S4, TIF file, 0.4 MB. Copyright © 2021 Villafraz et al. 2021 Villafraz et al. https://creativecommons.org/licenses/by/4.0/ This content is distributed under the terms of the Creative Commons Attribution 4.0 International license.

10.1128/mBio.00375-21.5 FIG S5 Subcellular localization of UGP in the presence or the absence of glucose. The ratios of glycosomal to cytosolic UGP were compared between the procyclic trypanosomes grown in the presence (+) and the absence (–) of glucose. The figure shows a Western blot of glycosomal (G) and cytosolic (C) fractions obtained after subcellular fractionation. Glycosomal (αPPDK) and cytosolic (αENO) markers are also shown. Download FIG S5, TIF file, 0.2 MB. Copyright © 2021 Villafraz et al. 2021 Villafraz et al. https://creativecommons.org/licenses/by/4.0/ This content is distributed under the terms of the Creative Commons Attribution 4.0 International license.

### Expression of glycosomal rUGP-GPDH rescues the lethality of the *^RNAi^*UGP mutant.

The *^EXP^*rUGP-GPDH construct (pHD1336-rUGP-GPDH), which produces an exclusively glycosomal rUGP, was introduced into the *^RNAi^*UGP-H10 cell line. Western blot analyses showed that native UGP was no longer detectable in the glycosomal and cytosolic fractions of the *^RNAi^*UGP/*^EXP^*rUGP-GPDH.i cell line, while the dying *^RNAi^*UGP.i cells still expressed residual amounts of UGP in the cytosol (Fig. 6B, right panel). The exclusive glycosomal subcellular localization of the recombinant rUGP-GPDH protein in the *^RNAi^*UGP/*^EXP^*rUGP-GPDH.i cell line observed by cellular fractionation (Fig. 6B) was confirmed by digitonin titration (Fig. 6C). In the context of the absence of cytosolic UGP, the viability of the *^RNAi^*UGP/*^EXP^*rUGP-GPDH.i cell line (Fig. 6A) strongly supported the hypothesis that the glycosomal pathway is functional. However, it be cannot excluded that residual expression of UGP in the cytosol is responsible for the growth of the *^RNAi^*UGP/*^EXP^*rUGP-GPDH.i cell line.

### The Δ*ugp*/*^EXP^*rUGP-GPDH cell line is viable.

**FIG 7:**
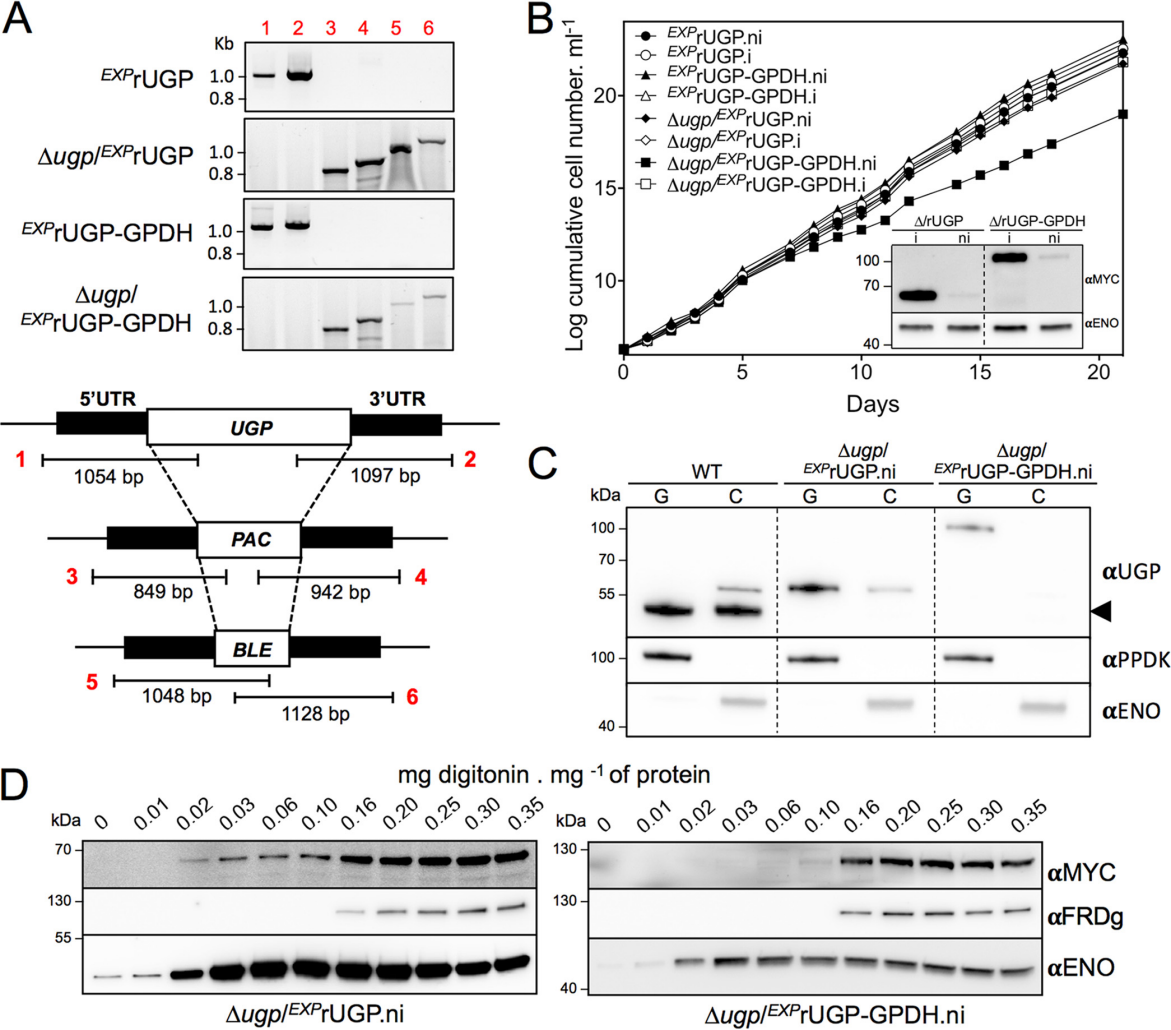
Production and functional analyses of Δ*ugp* cell lines. (A) PCR analysis of genomic DNA isolated from the parental (*^EXP^*rUGP and *^EXP^*rUGP-GPDH) and null mutant (Δ*ugp*/*^EXP^*rUGP and Δ*ugp*/*^EXP^*rUGP-GPDH) cell lines. Both knockout cell lines were obtained in the presence of tetracycline. Primers are designed on the basis of sequences flanking the 5′UTR and 3′UTR fragments used to target *UGP* gene depletion (black boxes) and on the open reading frame (ORF) of the *UGP* gene, as well as the puromycin (*PAC*) and phleomycin (*BLE*) resistance genes (white boxes). Δ*ugp*/*^EXP^*rUGP represents a control cell line expressing a recombinant UGP with a dual cytosolic and glycosomal localization. (B) Glycosomal (G) and cytosolic (C) fractions obtained after subcellular fractionation of the UGP null cell lines in the absence of tetracycline (5 days). The arrowhead highlights the native UGP only in parental (WT) cells. (C) The growth of the cell lines was followed during 21 days in the presence (.i) or the absence (.ni) of tetracycline. Western blot analyses with anti-MYC and anti-ENO (loading control) of the tetracycline-induced and noninduced (18 days after tetracycline removal) Δ*ugp*/*^EXP^*rUGP and Δ*ugp*/*^EXP^*rUGP-GPDH mutants are shown in the inset. (D) The UGP null cell lines were analyzed by digitonin titration 5 days after removal of tetracycline. A Western blot of supernatants confirmed the exclusive glycosomal localization of recombinant rUGP-GPDH with anti-MYC in the Δ*ugp*/*^EXP^*rUGP-GPDH cell line and the dual localization of rUGP in the Δ*ugp*/*^EXP^*rUGP cell line.

To confirm the functionality of the glycosomal pathway, rUGP-GPDH was expressed in the null UGP background (Δ*ugp*). Considering that UGP is an essential protein, knockout mutants were produced in two cell lines expressing tetracycline-inducible recombinant UGP, i.e., glycosomal/cytosolic rUGP and glycosomal rUGP-GPDH. The *UGP* alleles were replaced by the PAC and BLE markers after transfection with the recombinant plasmids expressing rUGP (Δ*ugp*/*^EXP^*rUGP) or rUGP-GPDH (Δ*ugp*/*^EXP^*rUGP-GPDH), in the presence of tetracycline to express the recombinant rUGP or rUGP-GPDH, respectively. Deletion of both *UGP* alleles was confirmed by PCR (Fig. 7A) and Western blotting (Fig. 7B). Tetracycline removal did not induce the death of the parasites (Fig. 7C), since the recombinant rUGP and rUGP-GPDH proteins were still expressed after 18 days in the absence of tetracycline (Fig. 7B, inset, and Fig. 7C). However, the growth of the Δ*ugp*/*^EXP^*rUGP-GPDH.ni cell line was slightly affected after tetracycline removal, which is consistent with the essential role of UGP. The absence of growth retardation for the Δ*ugp*/*^EXP^*rUGP.ni cell line, while the amounts of residual recombinant UGP were equivalent in the two cell lines (see Fig. 7C), might be interpreted as the cytosolic pathway having a higher efficiency than the glycosomal one. More importantly, the viability of the Δ*ugp*/*^EXP^*rUGP-GPDH.ni double mutant supports our hypothesis that the glycosomal pathway is functional.

These data, in agreement with the functional role of the glycosomal UGP, had to be confirmed by determining the subcellular localization of rUGP-GPDH in the Δ*ugp*/*^EXP^*rUGP-GPDH.ni cell line. After 5 days in the absence of tetracycline, the viable Δ*ugp*/*^EXP^*rUGP-GPDH.ni cell line expressed the recombinant rUGP-GPDH exclusively in the glycosomes (Fig. 7B to D, right panels). These data confirmed that the UDP-Glc/UDP-Gal biosynthetic pathway, which includes UGP, is active in the glycosomes. As expected, the MYC-tag rUGP showed dual glycosomal and cytosolic localizations in the Δ*ugp*/*^EXP^*rUGP.ni cell line (Fig. 7B to D).

### The glycosomal and cytosolic UGP-containing pathways are functional.

**FIG 8:**
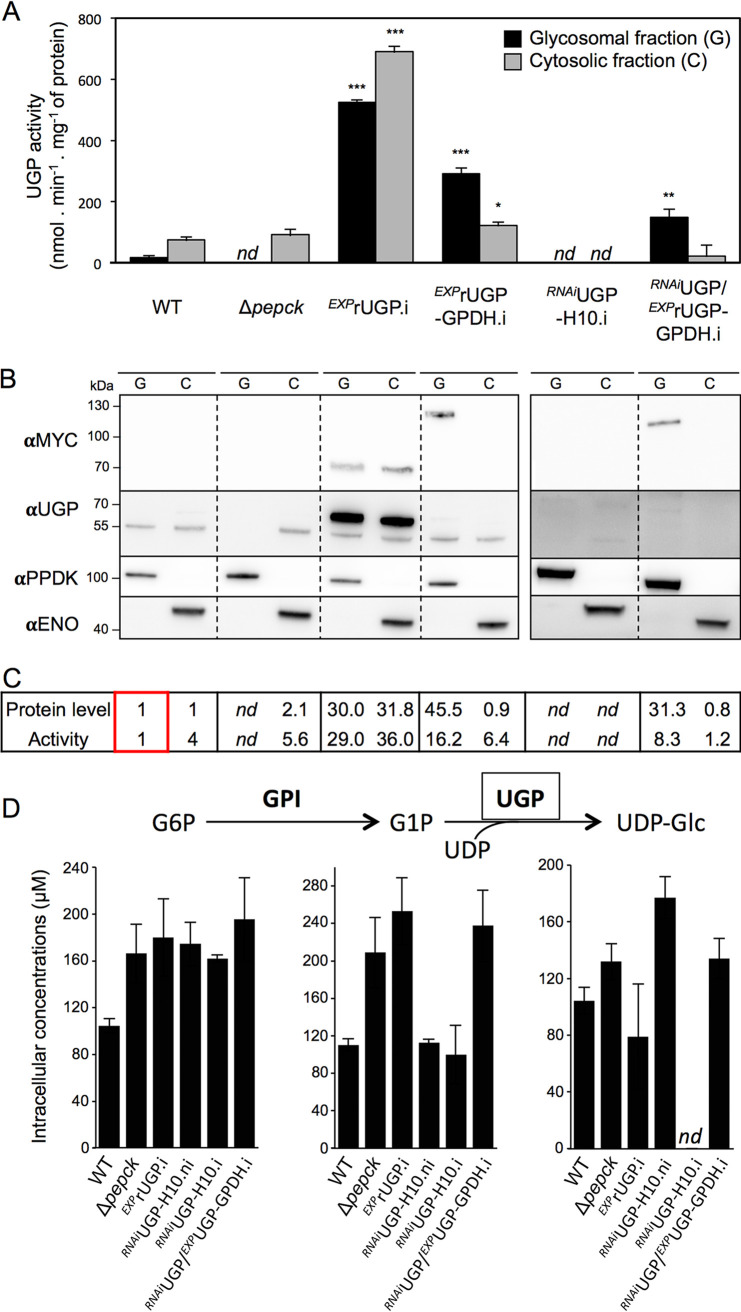
PCF produces UDP-Glc in glycosomes and the cytosol. (A) UGP activity was determined in enriched glycosomal and cytosolic fractions of the WT, Δ*pepck*, *^EXP^*rUGP.i, *^EXP^*rUGP-GPDH.i, *^RNAi^*UGP-H10.i, and *^RNAi^*UGP/*^EXP^*rUGP-GPDH.i cell lines (*n *= 3, SEM). The cytosolic and glycosomal UGP activities were normalized to the cytosolic malic enzyme and glycosomal glycerol kinase activities, respectively. This normalization consists of recalculating the UGP activities by considering that the malic enzyme and glycerol kinase activities are the same in the different cell lines. Significant differences between the WT and mutants are indicated for each compartment. ***, *P < *0.001; **, *P < *0.01; *, *P < *0.05. (B) Representative Western blots of the corresponding cell lines. Recombinant rUGP-GPDH was detected with anti-MYC antibody. Glycosomal (αPPDK) and cytosolic (αENO) markers are also shown. (C) Relative amounts of UGP (determined by Western blotting) and specific activity (average of 3 experiments). For these comparative analyses, the protein and activity levels detected in the glycosomal fraction of WT cells were used as references and given the arbitrary value of 1 (boxed values). (D) IC-HRMS analyses of intracellular metabolites (G6P, G1P, and UDP-Glc) collected from the indicated cell lines incubated in glucose-rich SDM79 medium. Only G6P, G1P, and UDP-Glc are shown in this figure; for other hexose phosphates and triose phosphates, see Fig. S6. *nd*, nondetectable.

To confirm the functionality of the glycosomal and cytosolic pathways involving UGP, cell lines expressing the native and/or recombinant UGP (i) in both subcellular compartments (wild type [WT], *^EXP^*rUGP.i, and *^EXP^*rUGP-GPDH.i), only in the cytosol (Δ*pepck*), (ii) only in the glycosomes (*^RNAi^*UGP/*^EXP^*rUGP-GPDH.i), or (iii) not at all (*^RNAi^*UGP-H10.i) were further analyzed (Fig. 8A to C). This included determining the expression levels of UGP in the glycosomal and cytosolic fractions by Western blotting and determination of enzymatic activities, as well as by quantifying intracellular metabolites, including the substrate (G1P) and the product (UDP-Glc) of the UGP enzymatic reaction, by a mass spectrometry-based metabolomics profiling approach (ion chromatography–high-resolution mass spectrometry [IC-HRMS]).

10.1128/mBio.00375-21.6 FIG S6 IC-HRMS analyses of intracellular metabolites collected from the indicated cell lines incubated in glucose-rich SDM79 medium. Download FIG S6, TIF file, 0.9 MB. Copyright © 2021 Villafraz et al. 2021 Villafraz et al. https://creativecommons.org/licenses/by/4.0/ This content is distributed under the terms of the Creative Commons Attribution 4.0 International license.

The specific activity of UGP (the ratio between the enzymatic activity and the relative amount of proteins detected by Western blotting) in the cytosolic fractions of *^EXP^*rUGP.i is ∼3.5-times lower than in the parental WT cells, suggesting that the C-terminal MYC tag affects UGP activity (Fig. 8C). Similarly, the native UGP shows a specific activity in the glycosomal fraction 4 times lower than in the cytosolic fraction of the parental cells, which suggests that the glycosomal sequestration of UGP affects its activity by a yet-unknown mechanism. These data provide a rational explanation for the growth retardation observed for the Δ*ugp*/*^EXP^*rUGP-GPDH.ni cell line, while the growth of the Δ*ugp*/*^EXP^*rUGP.ni cell line was not affected, although the amounts of residual recombinant UGP were equivalent in the two cell lines (Fig. 7C). We also confirmed that the coupling enzyme (UDP-Glc dehydrogenase) used in the UGP activity assays was not affected by the presence of the same amounts of the glycosomal or cytosolic samples (273 versus 245 mU · mg^−1^ of protein, respectively). The activity of the recombinant rUGPs, which is ∼30 times more expressed in the *^EXP^*rUGP.i line than the native UGP, was not affected in glycosomes, as the enzyme specific activities were similar in the glycosomal and the cytosolic fractions (Fig. 8C). It is also noteworthy that the UGP activity was detected in the cytosol of the *^RNAi^*UGP/*^EXP^*rUGP-GPDH.i line, while the native UGP was not detectable by Western blotting (Fig. 8B) and the recombinant rUGP-GPDH was exclusively glycosomal (Fig. 6C). This may be due to the rupture of a few glycosomes during the grinding step designed to disrupt primarily the plasma membrane.

To confirm the role of UGP subcellular localization in UDP-Glc production, we used mass spectrometry-based metabolomics to determine the intracellular amounts of G6P, G1P, and UDP-Glc (Fig. 8D), as well as other metabolites as controls (Fig. S6), in the cell lines mentioned above cultivated in SDM79 medium. This metabolomics approach was validated with the analysis of the Δ*pepck* cell line, in which the metabolic flux through the Gly3P/DHAP shuttle, used to maintain the glycosomal redox balance, has been reported to be increased in the absence of PEPCK (30). Indeed, the level of Gly3P is increased by ∼3 times in the Δ*pepck* mutant compared to levels in all the other cell lines analyzed (Fig. S6). Regarding the sugar nucleotide biosynthetic pathways, only UDP-Glc and UDP-GlcNAc were identified and quantified with this methodology (Fig. S6), and the levels of UDP-Glc detected (80 to 170 μM) were comparable to those previously reported for procyclic trypanosomes (110 to 540 μM) (39) (Fig. 8D). UDP-Glc was no longer detectable in the *^RNAi^*UGP.i cell line (Fig. 8D), which shows that UGP was the only enzyme producing UDP-Glc in PCF trypanosomes. It is also of note that UDP-Glc was detected in noninduced *^RNAi^*UGP cells at levels similar to those in parental cells, despite the ∼30-fold reduction of UGP protein levels (Fig. 6B), which shows that PCF trypanosomes express a large excess of UGP. Most importantly, UDP-Glc was produced in cells expressing UGP exclusively in the cytosol (Δ*pepck* cells) or in glycosomes (*^RNAi^*UGP/*^EXP^*rUGP-GPDH.i cells) at levels similar to those of WT cells, which confirms the functionality of the pathway in both subcellular compartments.

## DISCUSSION

Trypanosomatids are known to sequester a cascade of consecutive glycolytic enzymes into glycosomes, in addition to enzymes of other pathways, including those for gluconeogenesis, pentose phosphate, and sugar nucleotide biosynthesis (4, 40). In this study, we address three questions related to the glycosome biology by analyzing UGP, a key enzyme of sugar nucleotide biosynthesis involved in UDP-Glc synthesis. (i) The physiological role of this glycosomal pathway remains unknown since it is also present in the cytosol, the subcellular compartment where the biosynthesis of sugar nucleotides takes place in the other eukaryotes. (ii) The molecular mechanisms leading to the import of glycosomal enzymes lacking peroxisomal targeting signals (PTS1 or PTS2) have not yet been investigated in trypanosomatids. (iii) Mammalian peroxisomes multiply by the ER *de novo* route or by growth and division followed by protein import into newly produced organelles, but what about glycosomes? Here, we show that (i) the glycosomal pathway leading to the production of UDP-Glc and UDP-Gal is functional and is essential in PCF trypanosomes in the absence of the cytosolic pathway, (ii) UGP is imported into glycosomes by piggybacking on the PTS1-containing PEPCK, and (iii) PEPCK and UGP interact in only a few glycosomes, which may represent newly produced glycosomes competent for protein import.

### What is the role of sugar nucleotide biosynthesis in glycosomes?

The functionality of the glycosomal and cytosolic UGP-containing pathways was validated by the viability of mutants expressing UGP exclusively in glycosomes (*^RNAi^*UGP/*^EXP^*rUGP-GPDH.i cells) or the cytosol (Δ*pepck* cells) and the detection of UDP-Glc in both cell lines. This first direct evidence of a functional production of sugar nucleotides inside glycosomes raises two questions. First, how do *de novo*-synthesized UDP-Glc and UDP-Gal leave the glycosomes to reach the ER and Golgi apparatus, where they are required for protein glycosylation? The glycosomal membrane is considered to be impermeable to bulky metabolites, such as nucleotides, since the size limitation of the general peroxisomal diffusion pore is on the order of 400 Da (40, 41). Consequently, exchange of sugar nucleotides between the glycosomal and cytosolic compartments requires transporters. However, the only transporters known to be associated with the glycosomal membrane are the ABC transporters GAT1, GAT2, and GAT3, with GAT1 likely transporting acyl coenzyme A’s (acyl-CoAs) (42, 43), and proteomics analyses of glycosomal membrane fractions did not reveal additional candidates (44). Further work is certainly required to confirm the presence of such sugar nucleotide transporters in the glycosomal membrane. Second, what is the role of sugar nucleotide biosynthesis inside the glycosomes, since the cytosolic pathway is functional in the procyclic trypanosomes, as observed in all eukaryotes? UGP has also been localized in the Golgi apparatus, chloroplasts, and membrane fractions, as well as in cell walls, where it also provides UDP-Glc to produce glycoconjugates in plants and yeasts (45). Interestingly, the yeast UGP also shows a dual subcellular localization depending on phosphorylation at the N-terminal S11 residue, with the nonphosphorylated cytosolic and phosphorylated cell wall enzymes being involved in glycogenesis and cell wall glucan synthesis, respectively (21). All of these biosynthetic pathways require glycosyltransferases, which have not been detected in the glycosomal proteomes (46, 47) or in the repertoire of PTS-containing proteins (48). This supports the view that UDP-Glc and UDP-Gal are not produced in the glycosomes to feed glycosylation reactions inside glycosomes. Alternatively, glycosomal UDP-Glc may have a signaling role, as previously observed in animals and plants (49, 50).

### Piggybacking is a low-efficiency import process, as observed for UGP.

Piggybacking has been described as an import mechanism with relatively low efficiency in four out of five examples of physiological hetero-oligomer import into peroxisomes reported so far, i.e., superoxide dismutase (SOD1) (25) and lactate dehydrogenase (LDH) (26) in mammals and pyrazinamidase/nicotinamidase (PNC1) (29) and malate dehydrogenase 2 (Mdh2) (28) in yeast, which are coimported with the PTS-containing copper chaperone SOD1 (CCS), readthrough-extended LDH (LDHBx), glycerol-3-phosphate dehydrogenase (GPD1), and Mdh3, respectively. These four coimported proteins display dual peroxisomal and cytosolic localizations, with the majority remaining within the cytosol (28, 51). Similarly, approximately half of UGP remains in the cytosol. The reason for this relatively low import efficiency has been elucidated by the demonstration that the PST1 receptor (PEX5), required for peroxisomal import of PTS1-containing proteins, binds preferentially to monomers rather than to oligomers (52). Interestingly, weak protein-protein interactions are sufficient to support piggyback import. Indeed, blue native gels failed to show an interaction between the mammalian SOD and CCS partners (25), and synthetic substrates designed to evaluate the import of proteins showed dissociation constants (*K_d_*) differing by over 3 orders of magnitude, with even an apparent *K_d_* of ∼6 × 10^−3^ M allowing the detection of piggyback import (53). Despite several attempts, we did not observe any interaction between UGP and PEPCK using coimmunoprecipitation or native gels, suggesting that these interactions are weak and transient. In agreement with this weak interaction, PEPCK is in large excess compared to UGP, as illustrated by the ∼30-fold-higher enzymatic activity of PEPCK than of UGP (670 versus 20 mU · mg^−1^ of protein) (54) and the ∼100-fold-higher peptide counts for PEPCK than for UGP in proteomics analyses of glycosomal fractions from PCFs (see the PXD020190 data set in the PRIDE partner repository). In conclusion, our results support the role of hetero-oligomer import by piggybacking as an alternative route for import of glycosomal proteins, as described for peroxisomes of mammals and yeast. More importantly, the UGP/PEPCK association provides the first example of hetero-oligomeric import by piggybacking involving two proteins not functionally related, since PEPCK is involved in the maintenance of glycosomal redox and ATP/ADP balances, as well as gluconeogenesis (30, 35). Indeed, among the other known examples of piggybacking, CCS is the chaperone of SOD1 (25), LDH and LDHBx are encoded by the same gene (26), Mdh2 and Mdh3 are Mdh isoforms (28), and the PST1-containing phosphatase B subunit and phosphatases A/C subunits form an heterotrimeric enzymatic complex (27); however, the peroxisomal functions of PNC1 and GPD1 are unknown (29).

### UGP and PEPCK interact only transiently upon their import into newly produced import-competent glycosomes.

Since the formation of the UGP/PEPCK heterodimer may occur mainly during UGP import into the organelle, the analysis of UGP/PEPCK interactions using the PLA approach provides new insights into glycosomal import of proteins and multiplication of the organelles. In mammalian cells, peroxisomes multiply by the *de novo* ER route and by growth and division. The latter case involves an asymmetric process generating new peroxisomes via formation of a membrane compartment and subsequent import of newly synthesized matrix proteins (55–57). Indeed, overexpression of the membrane peroxin Pex11pβ resulted in the formation in mammalian cells of preperoxisomal membrane structures composed of mature globular domains and tubular extensions, the latter being maturated by import of matrix proteins (56). Equivalent clusters of tubular glycosomal membranes were also observed by overexpressing Pex11 in T. brucei (58), and clusters of elongated glycosomes have more recently been observed in BSF trypanosomes by whole-cell reconstruction using three-dimensional (3D) electron microscopy (59). In addition, T. brucei expresses Fis1 and Dpl1, two key proteins involved in the fission of newly produced peroxisomes in other eukaryotes (60–62). Overall, these observations confirm that glycosomes multiply by growth and division, as observed for the mammalian peroxisomes. This also implies that the new peroxisomes/glycosomes produced by growth and division are the most competent organelles for protein import and that they represent only a limited fraction of the organelle population, supporting the heterogeneity observed before among the peroxisomal (63) and glycosomal (33) populations. We thus propose that the structures showing close UGP/PEPCK proximity by PLA correspond to newly produced import-competent glycosomes. Considering that (i) PEPCK and UGP physically interact mainly during import at the glycosomal membrane because of their weak and transient interaction, (ii) that only up to 10 dots per cell correspond to physical proximity between PEPCK and UGP, with most cells containing 2 to 5 dots (Fig. 2), while PEPCK and UGP appear localized in almost all, if not all, glycosomes (Fig. 1E and Fig. S1), and (iii) that the number of glycosomes was estimated to be 60 to 65 per G_1_ trypanosome cell (59, 64), one could consider that the 3 to 10% of the organelles showing UGP/PEPCK interaction by PLA are newly produced glycosomes importing the matrix proteins, including PEPCK and UGP, in this context.

## MATERIALS AND METHODS

### Trypanosomes and cell cultures.

The procyclic form of T. brucei EATRO1125.T7T (TetR-HYG T7RNAPOL-NEO, where TetR stands for tetracycline resistance, HYG is hygromycin, POL is polymerase, and NEO is neomycin) was cultured at 27°C in SDM79 medium containing 10% (vol/vol) heat-inactivated fetal calf serum, 5 μg · ml^−1^ hemin (65), 25 μg · ml^−1^ hygromycin, and 10 μg · ml^−1^ neomycin. Alternatively, the cells were cultivated in a glucose-free medium derived from SDM79, called SDM79-GlcFree (37). The bloodstream form of T. brucei 427 90-13 (TetR-HYG T7RNAPOL-NEO) was cultured at 37°C in Iscove’s modified Dulbecco’s medium (IMDM) supplemented with 10% (vol/vol) heat-inactivated fetal calf serum (FCS), 0.25 mM β-mercaptoethanol, 36 mM NaHCO_3_, 1 mM hypoxanthine, 0.16 mM thymidine, 1 mM sodium pyruvate, 0.05 mM bathocuproine, and 2 mM l-cysteine (66). Cells were transfected as previously described (67). Overexpression and RNAi cell lines were induced with tetracycline (1 μg · ml^−1^). Growth was monitored by daily cell counting with the cytometer Guava EasyCyte.

### Expression of MYC-tagged UGP, TY-tagged UGP, eGFP-PEPCK truncations, and TY-tagged PEPCK.

The *UGP* gene (Tb927.10.13130) was cloned using the In-Fusion cloning system (Clontech) at the HindIII-NdeI restriction sites of pLew100-X-MYC, which was designed for expression of recombinant protein tagged at the C-terminal extremity with 3 MYC epitopes (modified from reference 68). The EATRO1125.T7T parental cell line and the Δ*pepck* (30), TY-PEPCK, and Δ*pepck*/TY-PEPCK cell lines were transfected with the pLew100-UGP-MYC tetracycline-inducible plasmid, and cells were selected in SDM79 containing phleomycin (5 μg · ml^−1^). The *UGP* gene was also *in situ* tagged at the N-terminal or C-terminal extremity, as previously described (69). The *TY-UGP* gene is flanked by the aldolase 5′ untranscribed region (5′UTR) and the UGP 3′UTR, while the *UGP-TY* gene is flanked by the UGP 5′UTR and the aldolase 3′UTR. Briefly, the DNA sequence encoding 10×TY1 tag and blasticidin (BLA) resistance cassette was amplified from the pPOTv7-10×TY1 vector using long primers (see Table S1 in the supplemental material) that incorporate a 5′ overhang of 80 nucleotides (nt) homologous to the *UGP* gene and its UTR. For the production of truncated UGP versions tagged with 10×TY1 at their C-terminal extremity, the forward primers were designed within the *UGP* gene extension to produce proteins containing the first 66 (1 to 66), 124 (1 to 124), 173 (1 to 173), and 226 (1 to 226) N-terminal residues. The PCR products were precipitated with ethanol before being used for transfection, and cells were selected in SDM79 containing blasticidin (20 μg · ml^−1^). We also expressed truncated versions of a recoded UGP (rUGP) (Fig. S7) lacking either the first 66 or the first 124 residues fused to the 10×TY1 tag at their C-terminal extremity. The PCR fragments corresponding to a complete or truncated *rUGP* gene fused to the TY tag and blasticidin cassette from pPOTv7 were obtained by overlapping PCR and cloned into pGEM-T. Cells were transfected with 10 μg of plasmid digested with NotI. For expression of truncated eGFP-PEPCK versions, the Δ*pepck* cell line (30) was transfected with the pLew100 tetracycline-inducible plasmid containing truncated versions of PEPCK fused at the N-terminal extremity to eGFP to increase the stability of the truncated recombinant proteins. PCR fragments corresponding to the truncations of PEPCK at residues 140, 180, 214, and 321 were inserted between the XhoI and XbaI restriction sites of the pLew100-eGFPX plasmid using the In-Fusion cloning system (Clontech). The PEPCK gene was also *in situ* tagged at the N terminus, as described above, with the *TY-PEPCK* gene flanked by the aldolase 5′UTR and the PEPCK 3′UTR.

10.1128/mBio.00375-21.7 FIG S7 Sequence alignment of the recoded UGP sequence (rUGP) and native UGP. The residues modified to change the coding sequence without affecting the coded amino acid residue are shaded. For cloning purposes, the last nucleotide from the HindIII restriction site at position 1276 was modified (shaded T in UGP and A in rUGP). Download FIG S7, TIF file, 0.7 MB. Copyright © 2021 Villafraz et al. 2021 Villafraz et al. https://creativecommons.org/licenses/by/4.0/ This content is distributed under the terms of the Creative Commons Attribution 4.0 International license.

10.1128/mBio.00375-21.8 TABLE S1 PCR primer sequences and features. P, position in the gene; Fw, forward primer; Rv, reverse primer; RE, restriction sites (underlined). pPOT annealing sequences are shown in lowercase letters; cell line names are in square brackets. Download Table S1, DOCX file, 0.1 MB. Copyright © 2021 Villafraz et al. 2021 Villafraz et al. https://creativecommons.org/licenses/by/4.0/ This content is distributed under the terms of the Creative Commons Attribution 4.0 International license.

### Production of recombinant glycosomal UGP proteins.

To target UGP exclusively to the glycosomes, the recoded recombinant *UGP* (*rUGP*) (Fig. S7) gene was inserted in the pHD1336 expression vector (42). For this purpose, the rUGP was fused at its C-terminal extremity to a 3×MYC tag followed by (i) the sequence encoding the last 12 C-terminal residues of the glycosomal fumarate reductase (*FRDg*) gene, which contains a PTS1 (rUGP-FRDgPST1), (ii) the full-length PTS1-containing glycosomal phosphoglycerate (*PGKc*) gene (rUGP-PGKc*), and (iii) the full-length PTS1-containing glycerol-3-phosphate dehydrogenase (*GPDH*) gene (rUGP-GPDH). The K215 residue, essential for PGK activity (36), was replaced by alanine. In order to increase the net charge of residues at the C terminus, which is a major determinant of peroxisomal import efficiency (70), we modified one residue in the C-terminal extremity of PGK (TL**R**NRW-SSL instead of TL**S**NRW-SSL) and of GPDH (PA**R**PRT-SKM instead of PA**L**PRT-SKM). The pHD1336-rUGP-FRDgPST1 plasmid, provided by the GeneCust Company, was used for cloning the synthesized genes (GeneCust) PGKc* and *GPDH* in the MluI-BamHI restriction sites. The EATRO1125.T7T parental cell line was transfected, and cells were selected in SDM79 containing blasticidin (20 μg · ml^−1^).

### Inhibition of *UGP* gene expression.

The inhibition of UGP expression by RNAi was achieved by expression of stem-loop “sense/antisense” RNA molecules targeting a 537-bp fragment of the *UGP* gene introduced into the pLew100 tetracycline-inducible expression vector. A PCR-amplified 579-bp fragment, containing the antisense UGP sequence was inserted between HindIII and BamHI restriction sites of the pLew100 plasmid. Then, the separate 537-bp PCR-amplified fragment containing the sense *UGP* sequence was inserted upstream of the antisense sequence, using HindIII and XhoI restriction sites. The resulting plasmid, pLew-UGP-SAS, contains a sense and antisense version of the UGP fragment separated by a 42-bp fragment. The *^RNAi^*UGP and *^RNAi^*UGP/*^EXP^*rUGP-GPDH mutants were generated by transfecting the EATRO1125.T7T and *^RNAi^*UGP cell lines with the pLew-UGP-SAS plasmid and the pHD1336-rUGP-GPDH plasmid, respectively. Transfected cells were selected in SDM79 medium containing hygromycin (25 μg · ml^−1^), neomycin (10 μg · ml^−1^), and phleomycin (5 μg · ml^−1^), with addition of blasticidin (20 μg · ml^−1^) for the *^RNAi^*UGP/*^EXP^*rUGP-GPDH cell line.

### Production of UGP null mutants.

Replacement of the *UGP* gene by the phleomycin and puromycin resistance markers via homologous recombination was performed with DNA fragments containing the resistance marker gene flanked by the UTR sequences. Briefly, an HpaI DNA fragment containing the *PAC* or *BLE* resistance marker gene preceded by the UGP 5′UTR fragment (522 bp) and followed by the UGP 3′UTR fragment (526 bp) was cloned into the pGEM-T plasmid. The UGP knockout mutants were generated in the *^EXP^*rUGP-GPDH and *^EXP^*rUGP cell lines in the presence of tetracycline. The *^EXP^*rUGP cell line was generated by transfecting the EATRO1125.T7T parental cell line with the pHD1336 vector expressing the *rUGP* sequence followed by a MYC tag sequence under the control of tetracycline. Transfected cells were selected in SDM79 medium containing blasticidin (20 μg · ml^−1^), phleomycin (5 μg · ml^−1^), puromycin (1 μg · ml^−1^), and tetracycline (1 μg · ml^−1^). The selected cell lines *rUGP*::*GPDH^Ti^-BLA TetR-HYG T7RNAPOL-NEO* Δ*ugp*::*PAC/*Δ*ugp*::*BLE* and *rUGP^Ti^-BLA TetR-HYG T7RNAPOL-NEO* Δ*ugp*::*PAC/*Δ*ugp*::*BLE* are called Δ*ugp/^EXP^*rUGP and Δ*ugp*/*^EXP^*rUGP-GPDH, respectively.

### Preparation of glycosomal and cytosolic fractions.

Cell homogenates were obtained by grinding prewashed cells with silicon carbide (200 mesh) in STE buffer (25 mM Tris, 1 mM EDTA, 250 mM sucrose, pH 7.8) (71) supplemented with the Complete EDTA-free protease inhibitor cocktail (Roche). The cells were microscopically checked for at least 90% disruption. The lysates were diluted in 7 ml of STE and centrifuged at 1,000 × *g* and then at 5,000 × *g* for 10 min each time at 4°C. The supernatants were centrifuged at 42,000 × *g* for 10 min at 4°C to yield the glycosome-enriched pellets and the cytosolic fractions (supernatants). The glycosomal pellets were washed once with 1 ml of STE before centrifugation at 42,000 × *g* for 10 min at 4°C and resuspension in 0.2 ml of STE. Equivalent amounts of protein from glycosomal and cytosolic fractions were analyzed by Western blotting.

### Digitonin permeabilization.

Trypanosomes were washed two times in cold phosphate-buffered saline (PBS) and resuspended at 10 mg of protein per ml in STE buffer supplemented with 150 mM NaCl and protease inhibitors. Cell aliquots (100 μl) were incubated with increasing quantities of digitonin (Sigma) for 4 min at 25°C, before centrifugation at 14,000 × *g* for 2 min. The supernatants were analyzed by Western blotting.

### Cell fractionation by hypotonic lysis.

BSF and PCF parasites (2 · 10^8^) were washed in PBS and hypotonically lysed in the presence of protease inhibitors by incubating them in 5 mM Na_2_HPO_4_, 0.3 mM KH_2_PO_4_ for 30 min at 4°C before centrifugation at 14,000 × *g* for 15 min. The pellet was solubilized in 2% SDS, and both the supernatant and the pellet were analyzed by Western blotting.

### Immunofluorescence and proximity ligation assay (PLA).

Cells were washed twice with PBS and then treated (+) or not treated (–) with 0.04 mg of digitonin per mg of protein for 4 min at 25°C. After centrifugation at 14,000 × *g* for 2 min and being washed, the cellular pellets were resuspended in PBS and fixed with 4% paraformaldehyde (PFA) for 10 min at room temperature. The cells were spread on poly-l-lysine-coated slides and permeabilized with 0.05% Triton X-100. After incubation in PBS containing 4% bovine serum albumin (BSA) overnight, cells were incubated for 45 min with primary antibodies (Table S2), washed with PBS, and incubated for 45 min with secondary antibodies (Table S2). Slides were washed and mounted with SlowFade Gold (Molecular Probes). Images were acquired with MetaMorph software on a Zeiss Imager Z1 or Axioplan 2 microscope as previously described (72).

10.1128/mBio.00375-21.9 TABLE S2 Antibodies used in this study. Download Table S2, DOCX file, 0.01 MB. Copyright © 2021 Villafraz et al. 2021 Villafraz et al. https://creativecommons.org/licenses/by/4.0/ This content is distributed under the terms of the Creative Commons Attribution 4.0 International license.

*In situ* PLA was performed using the Duolink *In Situ* Red mouse/rabbit starter kit (Sigma-Aldrich) according to the manufacturer's recommendations. Briefly, PFA-fixed and Triton X-100-permeabilized cells were spread on slides as described above. The cells were blocked with Duolink blocking solution for 60 min at 37°C. Primary rabbit anti-MYC (1/1,000) and mouse anti-TY1 (1/5,000) antibodies were diluted in Duolink antibody diluent and incubated for 60 min at room temperature. The slides were washed for 10 min in wash buffer A and incubated with the PLUS and MINUS PLA probes for 60 min at 37°C. Ligation and amplification steps were performed according to the manufacturer’s instructions. After being washed, cells were blocked with PBS-4% BSA overnight. The cells where counterstained with mouse anti-PPDK (αPPDK) (67). Slides were mounted in Duolink *in situ* mounting medium with DAPI (4′,6-diamidino-2-phenylindole). Images were acquired as described for immunofluorescence and analyzed using ImageJ. Cells were counted manually using cell counter ImageJ plugin.

### BN-PAGE.

Cells (10^8^) were washed in PBS and resuspended in SoTE (0.6 M sorbitol, 2 mM EDTA, 20 mM Tris-HCl, pH 7.5) (73). Cells were incubated with 0 or 0.16 mg of digitonin per mg of protein for 4 min at 25°C before centrifugation at 14,000 × *g* for 2 min. The supernatants containing both the cytosolic and glycosomal proteins were analyzed by blue native PAGE (BN-PAGE) on a precast (3 to 12%) Bis-Tris polyacrylamide gel (Invitrogen) according to standard methods.

### Western blot analyses.

Total protein extracts (5 · 10^6^ cells), glycosomal and cytosolic fractions, or supernatants obtained after digitonin treatment were separated by SDS-PAGE (10%) and immunoblotted on Trans-Blot Turbo midi size polyvinylidene difluoride (PVDF) membranes (Bio-Rad) (74). Immunodetection was performed as described previously (74, 75) using the primary antibodies and conditions summarized in Table S2. Revelation was performed using the Clarity Western enhanced-chemiluminescence (ECL) substrate as described by the manufacturer (Bio-Rad). Images were acquired and analyzed with the ImageQuant LAS 4000 luminescent image analyzer.

### UGP activity assay.

The UGP activity in total lysates and aliquots of glycosomal and cytosolic fractions was measured as previously described (22). For normalization of the UGP activities, the malic enzyme activity was determined on the total cell extracts and the cytosolic fractions, as described before (76). For normalization of the UGP activities in glycosomal extracts, the glycerol kinase activity was determined as described before (77). The PEPCK activity was measured in total lysates as previously described (54).

### Label-free quantitative proteomics.

Enriched glycosomal fractions were loaded on a 10% acrylamide SDS-PAGE gel, and proteins were visualized by colloidal blue staining. The steps of sample preparation, protein digestion, and liquid chromatography-mass spectrometry (LC-MS) parameters used for nanoscale LC-tandem MS (nanoLC-MS/MS) analysis on a Q-Exactive mass spectrometer were previously described (78). For protein identification, the SEQUEST HT and Mascot 2.4 algorithms through Proteome Discoverer 1.4 software (Thermo Fisher Scientific Inc.) were used for protein identification in batch mode by searching against a Trypanosoma brucei protein database (11,119 entries, release 46). This database was downloaded from the http://tritrypdb.org website. Two missed enzyme cleavages were allowed. Mass tolerances in MS and MS/MS were set to 10 ppm and 0.02 Da. The oxidation of methionine, acetylation of lysine, and deamidation of asparagine and glutamine were searched as dynamic modifications. Carbamidomethylation on cysteine was searched as a static modification. Peptide validation was performed using the Percolator algorithm (79), and only “high-confidence” peptides corresponding to a 1% false-discovery rate (FDR) at the peptide level were retained. Raw LC-MS/MS data were imported in Progenesis QI (version 2.0; Nonlinear Dynamics, a Waters Company) for feature detection, alignment, and quantification. All sample features were aligned according to retention times by manually inserting up to 50 landmarks, followed by automatic alignment, to maximally lay over all the two-dimensional (*m/z* and retention time) feature maps. Singly charged ions and ions with a higher charge states than 6 were excluded from analysis. All remaining features were used to calculate a normalization factor for each sample that corrects for experimental variation. Peptide identifications (with an FDR of <1%) were imported into Progenesis. Only nonconflicting features and unique peptides were considered for calculation of quantification at the protein level. A minimum of two peptides matched to a protein was used as the criterion for identification as a differentially expressed protein.

### MS analyses of intracellular metabolites by IC-HRMS.

Parental and mutant cell lines grown in SDM79 medium were collected on filters by fast filtration preparation (2 · 10^7^ cells per filter), as described before (30). Metabolites were analyzed by liquid anion exchange chromatography on a Dionex ICS-5000+ reagent-free HPIC (Thermo Fisher Scientific, Sunnyvale, CA, USA) system coupled with a Thermo Scientific linear trap quadrupole (LTQ) Orbitrap Velos hybrid Fourier-transform (FT) mass spectrometer (FTMS; Thermo Fisher Scientific, San Jose, CA, USA). The metabolites were separated within 48 min using a linear gradient elution of KOH applied to an IonPac AS11 column (250 by 2 mm; Dionex) equipped with an AG11 guard column (50 by 2 mm; Dionex) at a flow rate of 0.35 ml · min**^−^**^1^. The column and autosampler temperature were 30°C and 4°C, respectively. The injected sample volume was 15 μl. Mass detection was carried out in the negative electrospray ionization (ESI) mode. The settings of the mass spectrometer were as follows: spray voltage was at 2.7 kV, capillary and desolvation temperatures were 350 and 350°C, respectively, and the maximum injection time was 50 ms. Nitrogen was used as the sheath gas (pressure, 50 units) and auxiliary gas (pressure, 5 units). The automatic gain control (AGC) was set at 1E6 for full-scan mode, with a mass resolution of 60,000 (at 400 *m/z*). Data acquisition was performed using Thermo Scientific Xcalibur software. The identification of metabolites relied upon matching accurate masses from an FTMS scan (mass tolerance of 5 ppm) with retention time using TraceFinder 3.2 software. The absolute levels of intracellular metabolites were quantified based on the isotope dilution mass spectrometry (IDMS) approach.

### Data availability.

The mass spectrometry proteomics data have been deposited in the ProteomeXchange Consortium (http://proteomecentral.proteomexchange.org) via the PRIDE partner repository (80) with the data set identifier PXD020190.
