# Supplementary figures and images for: The Trypanosome UDP-Glucose Pyrophosphorylase Is Imported by Piggybacking into Glycosomes, Where Unconventional Sugar Nucleotide Synthesis Takes Place
Source: mBio. 2021 May 28;12(3):e00375-21. doi: 10.1128/mBio.00375-21 (PMC8262884; doi:10.1128/mBio.00375-21)

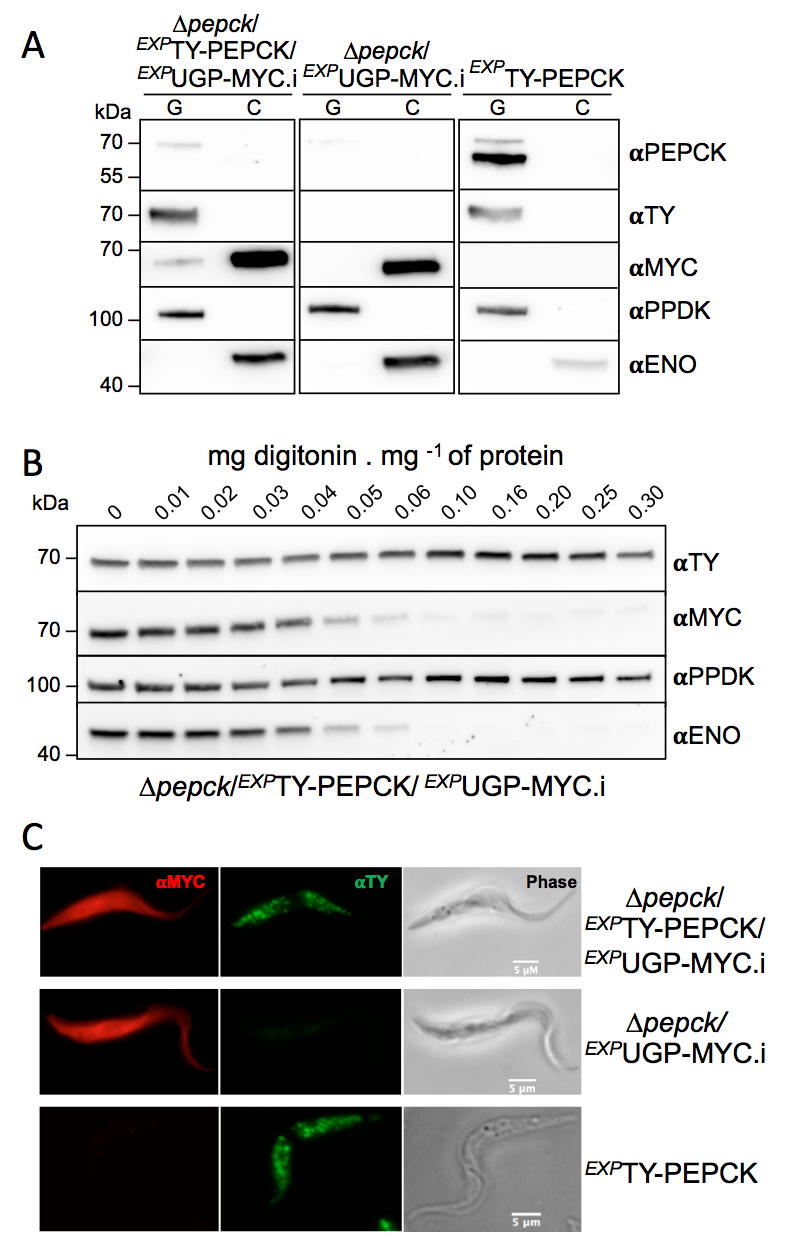

Supplement: FIG S1 [file mbio.00375-21-sf001.tif]

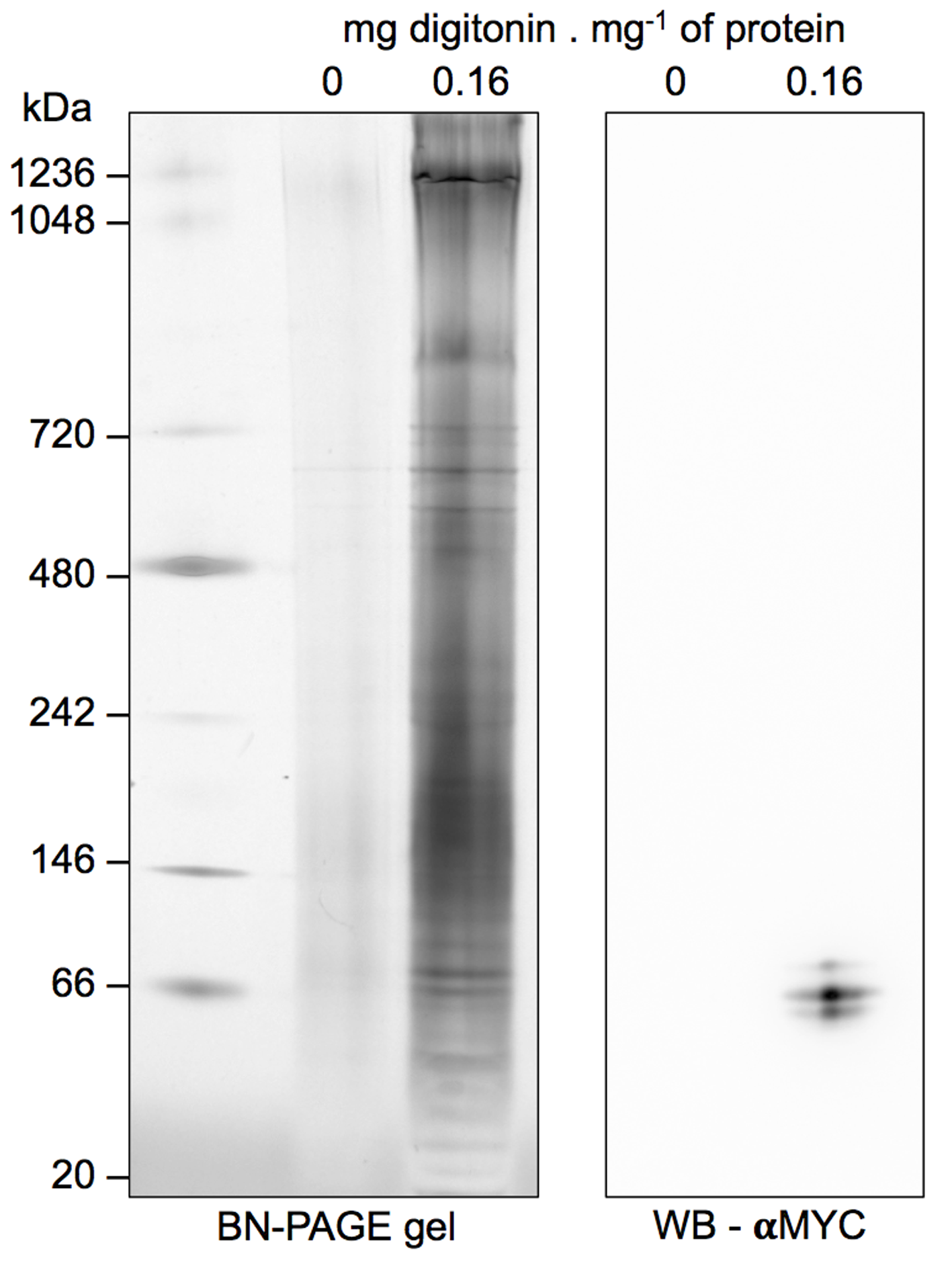

Supplement: FIG S2 [file mbio.00375-21-sf002.tif]

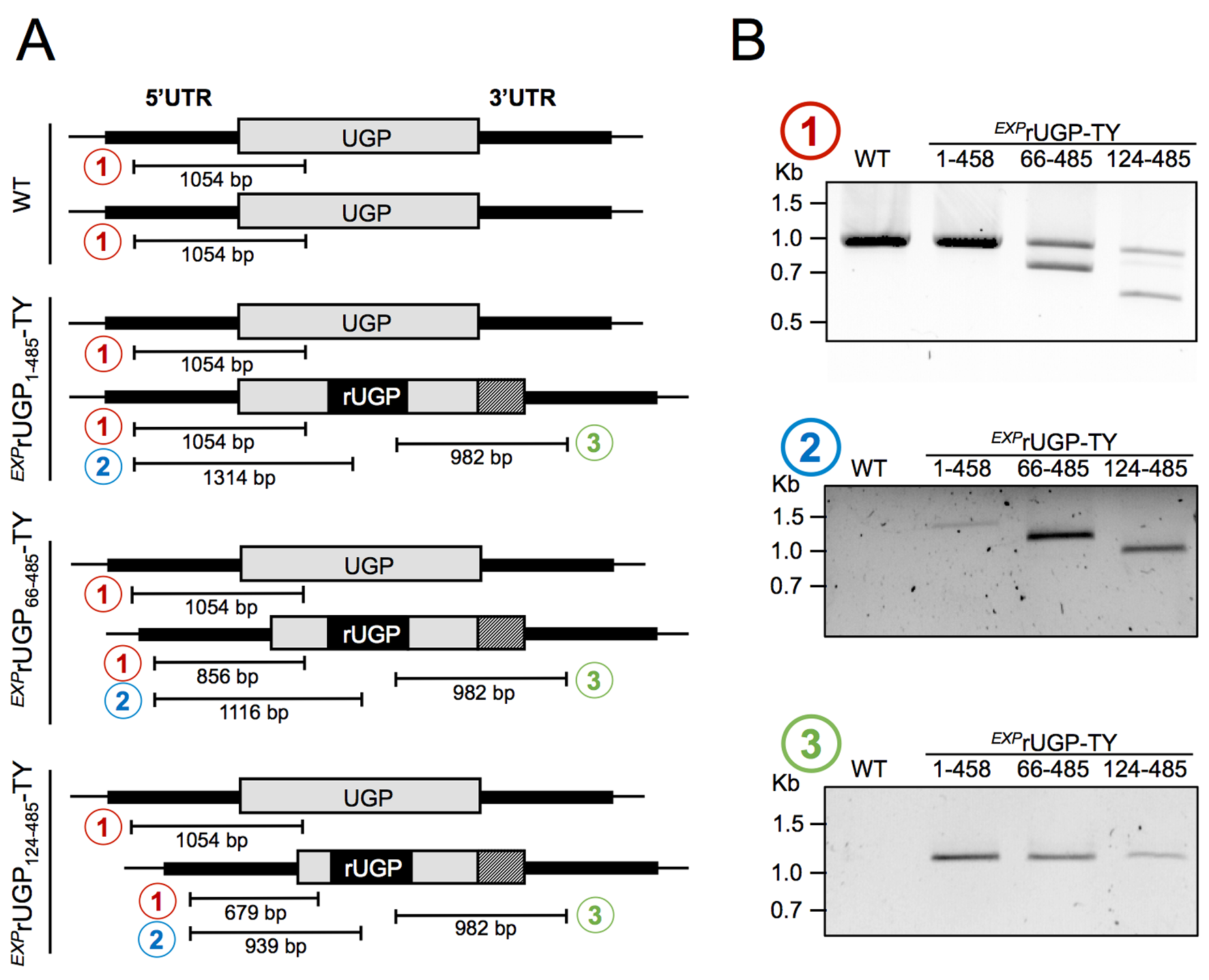

Supplement: FIG S3 [file mbio.00375-21-sf003.tif]

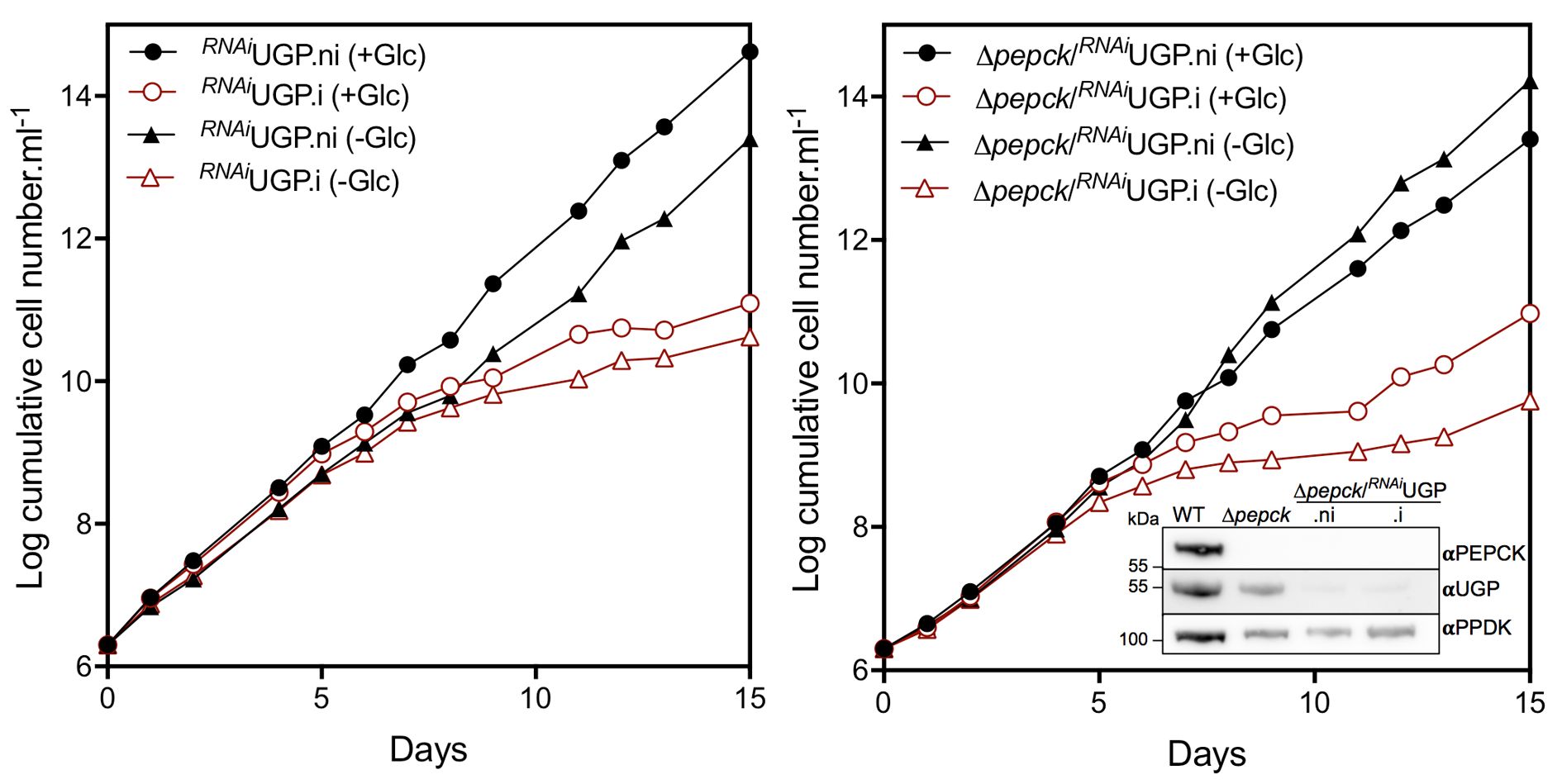

Supplement: FIG S4 [file mbio.00375-21-sf004.tif]

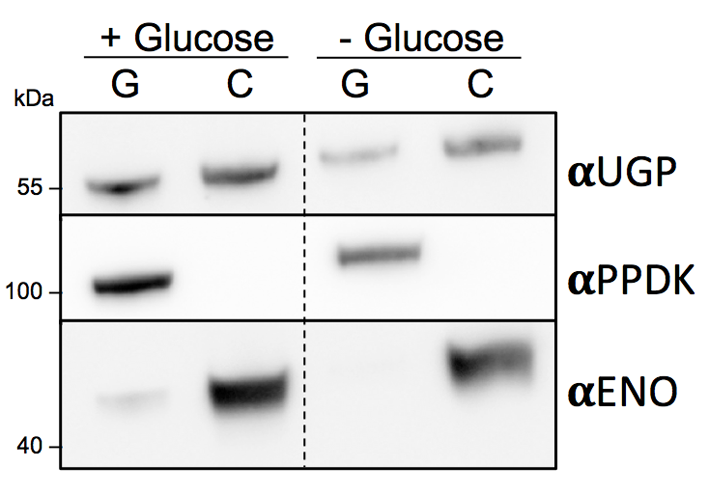

Supplement: FIG S5 [file mbio.00375-21-sf005.tif]

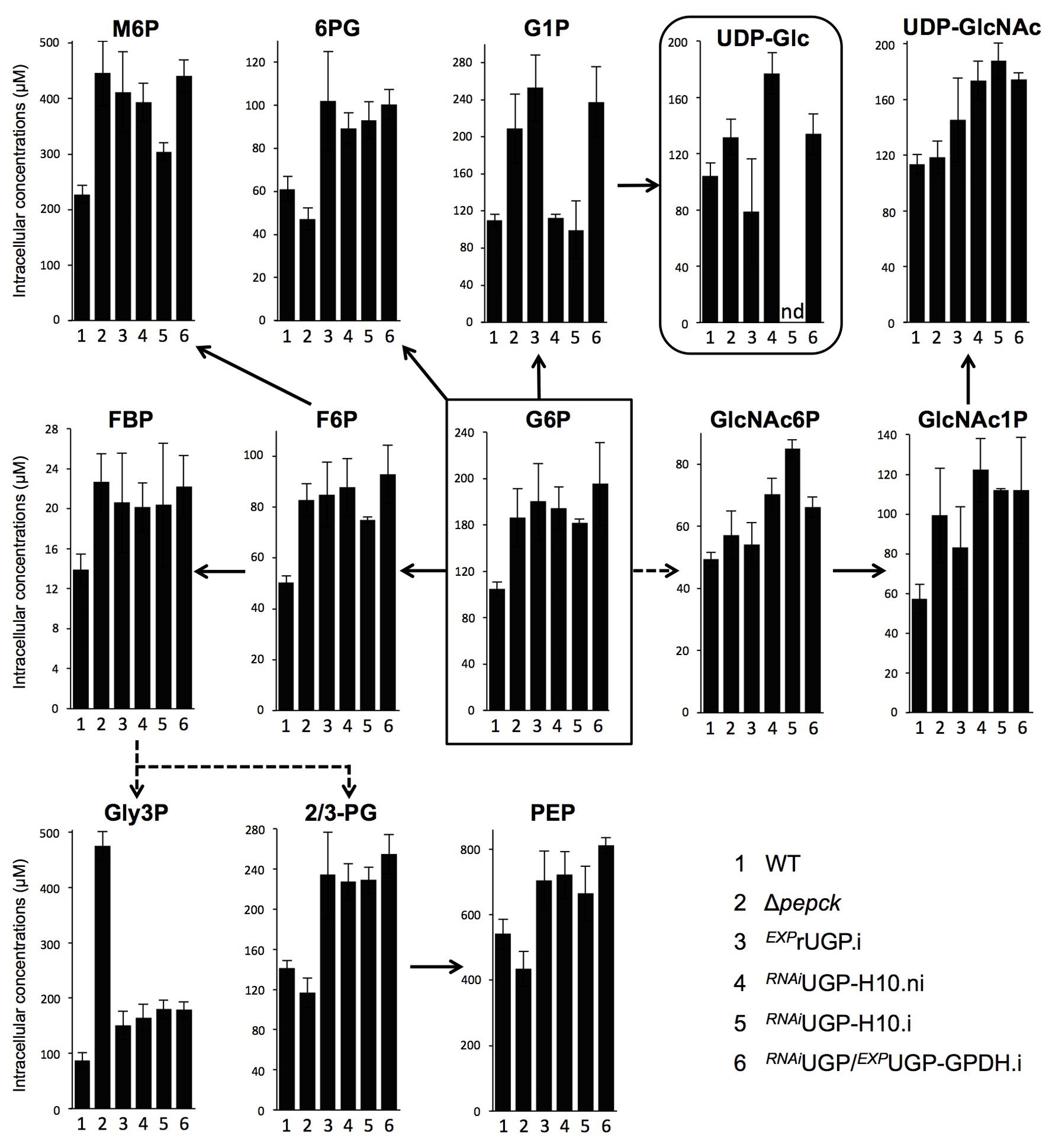

Supplement: FIG S6 [file mbio.00375-21-sf006.tif]

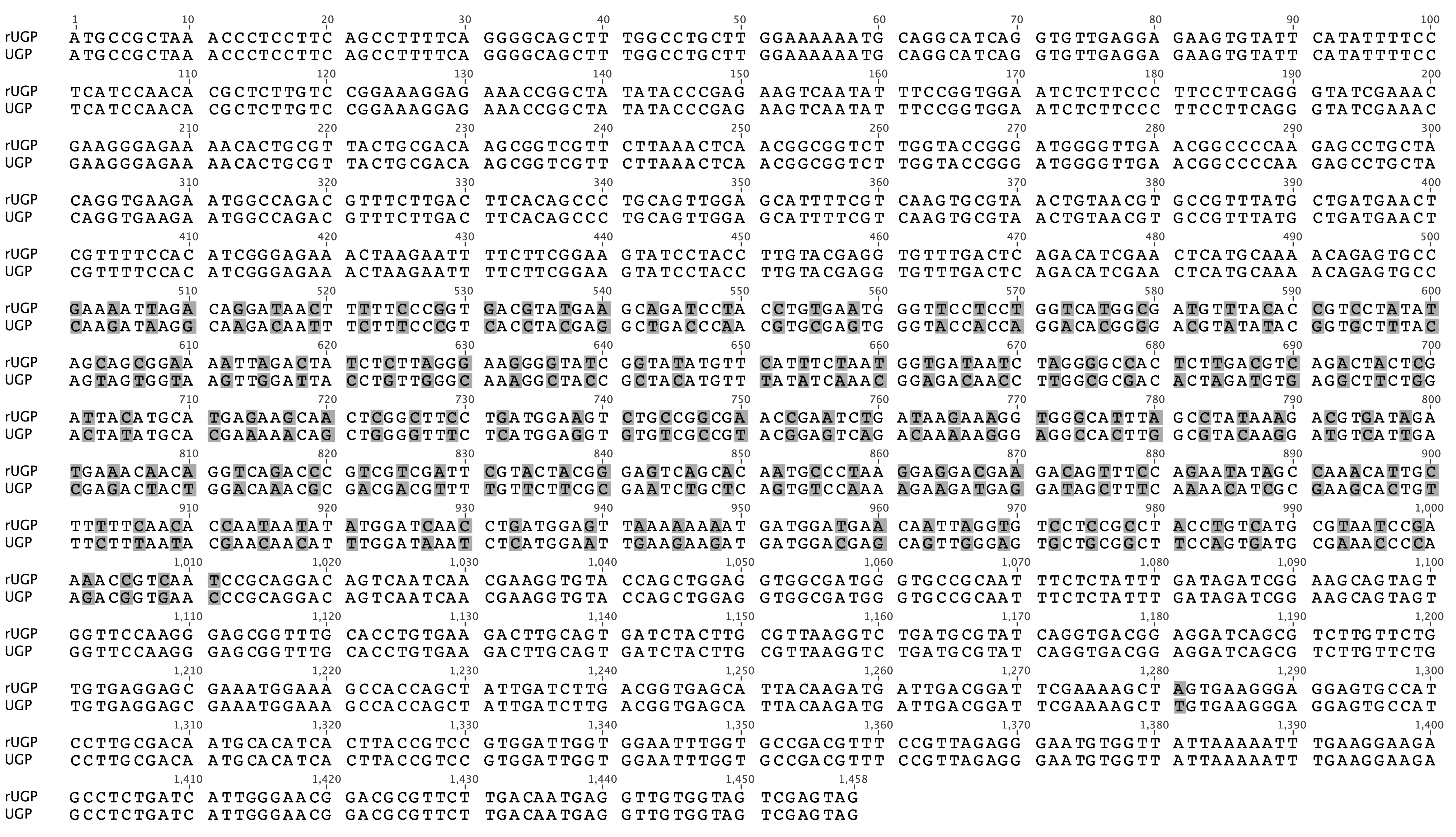

Supplement: FIG S7 [file mbio.00375-21-sf007.tif]
