# Supplementary material for: The Trypanosome UDP-Glucose Pyrophosphorylase Is Imported by Piggybacking into Glycosomes, Where Unconventional Sugar Nucleotide Synthesis Takes Place
Source: mBio. 2021 May 28;12(3):e00375-21. doi: 10.1128/mBio.00375-21 (PMC8262884; doi:10.1128/mBio.00375-21)
Supplement: TABLE S1 [file mbio.00375-21-st001.docx]

Table S1

|  | P |  | Primer name | Sequence 5'-3' | RE | Plasmid/  PCR product | Comments |
| --- | --- | --- | --- | --- | --- | --- | --- |
| PEPCK  Tb927.2.4210 |  | Fw | pPEPCKex1-5 | GCCCAATGATATCACAATGGCTCCTATAATTCACAA | EcoRV | pLew100 | Rescue [Δ*pepck*/*^EXP^*PEPCK] |
|  |  | Rv | pPEPCKex1-3 | CACGCCGGATCCCCTAACATATTAAAGACGCGAAGC | BamHI |  |  |
|  | 418 | Fw | pgfpPEPCKtc1-5 | GCTGTACAAGCTCGAGACAATGGACTATGTCATCTACAACG | XhoI | pLew100 | Expression GFP-PEPCK 140-525 [Δ*pepck*/*^EXP^*eGFP-PEPCK_140-525_] |
|  | 538 | Fw | GFP-PEPCK 180 | GCTGTACAAGCTCGAGGTCATTACGCGTACAATGTACGCTGGTGAAATGAAG | XhoI  MluI | pLew100 | Expression GFP-PEPCK 180-525 [Δ*pepck*/*^EXP^*eGFP-PEPCK_180-525_] |
|  | 640 | Fw | GFP-PEPCK 214 | GCTGTACAAGCTCGAGGCTAACACGCGTACAATGGGTGACGTGACGGTCTTC | XhoI  MluI | pLew100 | Expression GFP-PEPCK 214-525 [Δ*pepck*/*^EXP^*eGFP-PEPCK_214-525_] |
|  | 955 | Fw | pgfpPEPCKtc3-5 | GCTGTACAAGCTCGAGACAATGCTTTCGAAGGCTGTTGC | XhoI | pLew100 | Expression GFP-PEPCK 321-525 [Δ*pepck*/*^EXP^*eGFP-PEPCK_321-525_] |
|  |  | Rv | pgfpPEPCKex1-3 | ATTCACCGGTTCTAGACCTAACATATTAAAGACGCGAAGC | XbaI | pLew100 | Expression GFP-PEPCK  [Δ*pepck*/*^EXP^*eGFP-PEPCK_XXX-525_] |
|  |  | Fw | FWPEPCK-  TagNter | CTCTACAGCCGTCTTCAACAATTAACATTTTACGTTCTTATACTTATATATTTCGTTGTGTATTGCCCAATCATAACACAgtataatgcagacctgctgc |  | PCR  product | N-terminal tag [*^EXP^*TY-PEPCK] |
|  |  | Rv | RvPEPCK-  TagNter | GTCAGTTGGGAGTCCTTTTCAAGCTTAAGAGCCCACTCCACCAACTCGGGGGCGGTGAGATTCTTGTGAATTATAGGAGCactacccgatcctgatcc |  |  |  |
| UGP  Tb927.10.13130 |  | Fw | FwUGP3xMYC | TAAAATTCACAAGCTTACAATGCCGCTAAACCCTCCTTC | HindIII | pLew100 | Expression [*^EXP^*UGP-MYC, Δ*pepck*/*^EXP^*UGP-MYC, *^EXP^*TY-PEPCK/*^EXP^*UGP-MYC, Δ*pepck*/  *^EXP^*TY-PEPCK/*^EXP^*UGP-MYC] |
|  |  | Rv | RvUGP3xMYC | CGATCGGCCGCCCATATGCTCGACTACCACAACCTCATT | NdeI |  |  |
|  |  | Fw | TagNUGPFw | CAGAACGAACAGAAAAATACGCAAGCTCATAAGATCAGATACCTCGGAACGGTGTCAGTGTGTGCTGTTGTGTCACACGAgtataatgcagacctgctgc |  | PCR  product | N-terminal tag [*^EXP^*TY-UGP_1-485_] |
|  |  | Rv | TagNUGPRv | TCCTCAACACCTGATGCCTGCATTTTTTCCAAGCAGGCCAAAGCTGCCCCTGAAAAGGCTGAAGGAGGGTTTAGCGGCATactacccgatcctgatcc |  |  |  |
|  |  | Fw | TagCUGPFw | TGGTTATTAAAAATTTGAAGGAAGAGCCTCTGATCATTGGGAACGGACGCGTTCTTGACAATGAGGTTGTGGTAGTCGAGggttctggtagtggttcc |  | PCR  product | C-terminal tag  [*^EXP^*UGP_1-485_-TY] |
|  | 198 | Fw | UGP E66 | TCCGGAAAGGAGAAACCGGCTATATACCCGAGAAGTCAATATTTCCGGTGGAATCTCTTCCCTTCCTTCAGGGTATCGAAggttctggtagtggttcc |  | PCR  product | C-terminal tag – truncation 1-66  [*^EXP^*UGP_1-66_-TY] |
|  | 372 | Fw | N124 | GCCTGCTACAGGTGAAGAATGGCCAGACGTTTCTTGACTTCACAGCCCTGCAGTTGGAGCATTTTCGTCAAGTGCGTAACggttctggtagtggttcc |  | PCR  product | C-terminal tag – truncation 1-124 [*^EXP^*UGP_1-124_-TY] |
|  | 519 | Fw | N173 | AGTATCCTACCTTGTACGAGGTGTTTGACTCAGACATCGAACTCATGCAAAACAGAGTGCCCAAGATAAGGCAAGACAATggttctggtagtggttcc |  | PCR  product | C-terminal tag – truncation 1-173 [*^EXP^*UGP_1-173_-TY] |
|  | 678 | Fw | A226 | ACAGTAGTGGTAAGTTGGATTACCTGTTGGGCAAAGGCTACCGCTACATGTTTATATCAAACGGAGACAACCTTGGCGCGggttctggtagtggttcc |  | PCR  product | C-terminal tag – truncation 1-226 [*^EXP^*UGP_1-226_-TY] |
|  |  | Rv | TagCUGPRv | TGCAAGTACTTGTGTGTACCACACTTTCCTAACCTCCCCCCCTTTTCTCTTTAGATGTGACAGGTAAGCTGTTTTATCACccaatttgagagacctgtgc |  | PCR  product | C-terminal tag  [*^EXP^*UGP_1-XXX_-TY] |
|  |  | Fw | FwUGP-66-OL | CAGAACGAACAGAAAAATACGCAAGCTCATAAGATCAGATACCTCGGAACGGTGTCAGTGTGTGCTGTTGTGTCACACGAATGACGAAGGGAGAAAACACTGCG |  |  | Construction of plasmid pGEM®-T Easy-UGP-66-TY by Overlapping PCR |
|  |  | Rv | RvUGP-66-OL | ACCGGAACCACTACCAGAACCCTCGACTACCACAACCTCATTGTC |  |  |  |
|  |  | Fw | FwUGPcTag-OL | GACAATGAGGTTGTGGTAGTCGAGGGTTCTGGTAGTGGTTCCGGT |  |  |  |
|  |  | Rv | TagCUGPRv | TGCAAGTACTTGTGTGTACCACACTTTCCTAACCTCCCCCCCTTTTCTCTTTAGATGTGACAGGTAAGCTGTTTTATCACccaatttgagagacctgtgc |  |  |  |
|  |  | Fw | FwUGPrecComp  OL | CCATGGCGGCCGCGGCAGAACGAACAGAAAAATACGCAAGCTCATAAGATCAGATACCTCGGAACGGTGTCAGTGTGTGCTGTTGTGTCACACGAATGCCGCTAAACCCTCCTTCAGCC | SacII | pGEM®-T Easy | C-terminal tag 1-485 (Cloning rUGP in pGEM®-T Easy-UGP-66-TY) [*^EXP^*rUGP_1-485_-TY] |
|  | 198 | Fw | FwUGPrec66OL | CCATGGCGGCCGCGGCAGAACGAACAGAAAAATACGCAAGCTCATAAGATCAGATACCTCGGAACGGTGTCAGTGTGTGCTGTTGTGTCACACGAATGACGAAGGGAGAAAACACTGCG | SacII | pGEM®-T Easy | C-terminal tag 66-485 (Cloning rUGP-66 in pGEM®-T Easy-UGP-66-TY [*^EXP^*rUGP_66-485_-TY] |
|  | 372 | Fw | FwUGP124OL | CCATGGCGGCCGCGGCAGAACGAACAGAAAAATACGCAAGCTCATAAGATCAGATACCTCGGAACGGTGTCAGTGTGTGCTGTTGTGTCACACGAATGTGTAACGTGCCGTTTATGCTG | SacII | pGEM®-T Easy | C-terminal tag 124-458 (Cloning rUGP-124 in pGEM®-T Easy-UGP-66-TY [*^EXP^*rUGP_124-485_-TY] |
|  |  | Rv | RvUGPrec66OL | TTGTGTGTACCTCAAGCTTAGAACCGGAACCGGAACCACTACCAGAACCCTCGACTACCACAACCTCATTGTC | HindIII | pGEM®-T Easy | C-terminal tag rUGP  [*^EXP^*rUGP_XXX-485_-TY] |
|  |  | Fw | UGPo5'5' | ACATGAAGAATGGGAAGATATTGATG* |  |  | Confirm *in situ* tagging and ∆*ugp* 5’UTR (*) |
|  |  | Rv | UGPo5'3' | CGAGTTCATCAGCATAAACGG* |  |  |  |
|  |  | Rv | rUGPo5’3’ | CCATTAGAAATGAACATATACC |  |  |  |
|  |  | Fw | rUGPTYoFw | GTCATGCGTAATCCGAAAACC |  |  | Confirm *in situ* tagging |
|  |  | Rv | rUGPTYoRv | CGTACACAGCGGCATAGATTG |  |  |  |
|  |  | Fw | pUGP-S5 | GCAAAAAAGCTTGCCCAAGATAAGGCAAGACAATT | HindIII | pLew100 | Cassette sense RNAi  [*^RNAi^*UGP-H10, *^RNAi^*UGP-E4, *^RNAi^*UGP/*^EXP^*rUGP-GPDH] |
|  |  | Rv | pUGP-S3 | CTGTCCCTCGAGTTCACCGTCTTGGGGTTTCGCAT | XhoI |  |  |
|  |  | Fw | pUGP-AS3 | TGCGGCAAGCTTCGCCACCTCGAGCTGGTACACCTTCGTTGATTGAC | HindIII  XhoI | pLew100 | Cassette antisense RNAi  [*^RNAi^*UGP-H10, *^RNAi^*UGP-E4, *^RNAi^*UGP/*^EXP^*rUGP-GPDH] |
|  |  | Rv | pUGP-AS5 | AACAGAGGATCCAAGATAAGGCAAGACAATTTC | BamHI |  |  |
|  |  | Fw | PUGP5UTR-5 | GTGTGTCCATGGGTGAGTGTTAACGAACACACACTCACACACATA | NcoI  HpaI | pGEM®-T | Cassette 5'UTR ∆*ugp* [∆*ugp/^EXP^*rUGP, ∆*ugp/^EXP^*rUGP-GPDH] |
|  |  | Rv | PUGP5UTR-3 | AGGAGGCTCGAGCGGCATTCTAGAACACAACAGCACACACTGACA | XhoI  XbaI |  |  |
|  |  | Fw | PUGP3UTR-5 | AATGAGTCTAGAGTAGTCAAGCTTGTGATAAAACAGCTTACCTGTCAC | XbaI  HindIII | pGEM®-T | Cassette 3'UTR ∆*ugp*  [∆*ugp/^EXP^*rUGP, ∆*ugp/^EXP^*rUGP-GPDH] |
|  |  | Rv | PUGP3UTR-3 | TGTTTTCTCGAGAAAGGTGTTAACAAACACACACTGTTCCCAATT | XhoI  HpaI |  |  |
|  |  | Fw | UGPo3'5' | GGAAGCAGTAGTGGTTCCAAG |  |  | Confirm ∆*ugp* 3’UTR |
|  |  | Rv | UGPo3'3'' | GCTGACTAACACAAACCATGTATC |  |  |  |
