# Supplementary material for: The Trypanosome UDP-Glucose Pyrophosphorylase Is Imported by Piggybacking into Glycosomes, Where Unconventional Sugar Nucleotide Synthesis Takes Place
Source: mBio. 2021 May 28;12(3):e00375-21. doi: 10.1128/mBio.00375-21 (PMC8262884; doi:10.1128/mBio.00375-21)
Supplement: TABLE S2 [file mbio.00375-21-st002.docx]

Table S2

| Primary antibodies | | | |
| --- | --- | --- | --- |
| Name | Species | Dilution | Source/Ref |
| Anti-TY | Mouse | WB 1:5,000  IF 1:5,000 | BB2-Gift from K. Gull, Oxford, UK |
| Anti-MYC | Mouse | WB 1:1,000  IF 1:100 | 9E10-Gift from K. Ersfeld, Hull, UK |
| Anti-MYC | Rabbit | IF 1:1,000 | Sigma, C3956 |
| Anti-UGP | Rabbit | WB 1:100 | COVALAB  GYIPEKSIFPVES and RNGKPPAIDLDGEH peptides |
| Anti-PEPCK | Rabbit | WB 1:1,000 | Proteogenix  HDGTLDQADYEVYPG and TDLKQFNETTKELVT peptides |
| Anti-PPDK | Mouse | IF 1:1 | (81)(80) |
|  | Rabbit | WB 1:1,000 |  |
| Anti-ENO | Rabbit | WB 1:100,000 | Gift from P. Michels, Edinburgh, UK |
| Anti-ALD | Rabbit | WB 1:5,000  IF 1:1,000 | Gift from P. Michels, Edinburgh, UK |
| Anti-FRDg | Rabbit | WB 1:1,000 | (82) |
| Secondary antibodies | | | |
| Anti-mouse HRP | Goat | WB 1:5,000 | Bio-Rad |
| Anti-rabbit HRP | Goat | WB 1:10,000 | Bio-Rad |
| Anti-mouse Alexa Fluor 594 | Donkey | IF 1:100 | ThermoFisher |
| Anti-mouse Alexa Fluor 488 | Donkey | IF 1:100 | ThermoFisher |
| Anti-rabbit Alexa Fluor 594 | Donkey | IF 1:100 | ThermoFisher |
| Anti-rabbit Alexa Fluor 488 | Donkey | IF 1:100 | ThermoFisher |
